# Green-fruited *Solanum habrochaites* lacks fruit-specific carotenogenesis due to metabolic and structural blocks

**DOI:** 10.1093/jxb/erx288

**Published:** 2017-10-09

**Authors:** Himabindu Vasuki Kilambi, Kalyani Manda, Avanish Rai, Chaitanya Charakana, Jayram Bagri, Rameshwar Sharma, Yellamaraju Sreelakshmi

**Affiliations:** Repository of Tomato Genomics Resources, Department of Plant Sciences, University of Hyderabad, Hyderabad, India

**Keywords:** Carotenoid biosynthesis, carotenoid sequestration, fruit color, gene expression, proteome analysis, tomato clade

## Abstract

Members of the tomato clade exhibit a wide diversity in fruit color, but the mechanisms governing inter-species diversity of coloration are largely unknown. The carotenoid profiles, carotenogenic gene expression and proteome profiles of green-fruited *Solanum habrochaites* (SH), orange-fruited *S. galapagense*, and red-fruited *S. pimpinellifolium* were compared with cultivated tomato [*S. lycopersicum* cv. Ailsa Craig (SL)] to decipher the molecular basis of coloration diversity. Green-fruited SH, though it showed normal expression of chromoplast-specific *phytoene synthase1* and *lycopene β-cyclase* genes akin to orange/red-fruited species, failed to accumulate lycopene and β-carotene. The SH *phytoene synthase1* cDNA encoded an enzymatically active protein, whereas the *lycopene β-cyclase* cDNA was barely active. Consistent with its green-fruited nature, SH’s fruits retained chloroplast structure and PSII activity, and had impaired chlorophyll degradation with high pheophorbide *a* levels. Comparison of the fruit proteomes with SL revealed retention of the proteome complement related to photosynthesis in SH. Targeted peptide monitoring revealed a low abundance of key carotenogenic and sequestration proteins in SH compared with tomato. The green-fruitedness of SH appears to stem from blocks at several critical steps regulating fruit-specific carotenogenesis namely the absence of chloroplast to chromoplast transformation, block in carotenoid biosynthesis, and a dearth of carotenoid sequestering proteins.

## Teaser

The green-fruited *Solanum habrochaites* encodes a functional phytoene synthase 1 but fails to accumulate lycopene due to metabolic and structural blocks in chromoplast formation and carotenoid storage.

## Introduction

The color of fruits is one of the traits that is highly diverse across the angiosperms. Members of the family Solanaceae show great diversity in fruit color and within one genus, *Solanum*, color varies from green to black, orange/yellow and red (Knapp, 2002). This wide range of colors is imparted by the accumulation of pigments such as carotenoids, anthocyanins, and flavonoids (Seymour *et al.*, 2013). In the Solanaceae, anthocyanins impart fruit color in eggplant and carotenoids contribute to the color of tomato fruits (Dhar *et al.*, 2015). At the same time, many Solanaceous plants have green fruits that do not accumulate these pigments (Knapp, 2002).

Fruit coloration signifies the completion of the ripening process and involves the loss of chlorophylls and accumulation of pigments in a sink tissue/organelle. In tomato (*S. lycopersicum*), the ripening process is accompanied by the conversion of chloroplasts to chromoplasts, which store the carotenoid pigments. The regulatory switch needed for the transformation to chromoplasts is not known except in cauliflower. In cauliflower curds, the expression of the *Orange* (*Or*) gene likely mediates the transformation of plastids to chromoplasts (Li *et al.*, 2001; Lu *et al.*, 2006) as the heterologous expression of the *Or* gene induces chromoplast differentiation in potato tubers (Lopez *et al.*, 2008).

Members of the tomato clade display a wide diversity in fruit color making it an excellent model group for examining the molecular basis of diversity in carotenogenesis (Bauchet and Causse, 2012). Most members of the tomato clade are green-fruited, excepting *S. galapagense* and *S. cheesmaniae* with orange fruits and *S. pimpinellifolium* and tomato with red fruits. Though green-fruited species *per se* do not accumulate lycopene, analysis of the introgression lines derived from species such as *S*. *hirsutum* (Bernacchi *et al.*, 1998), *S. peruvianum* (Fulton *et al.*, 1997), *S. parviflorum* (Fulton *et al.*, 2000), and *S. pennellii* (Liu *et al.*, 2003) revealed several quantitative trait loci (QTLs) that influence lycopene/ β-carotene accumulation in tomato.

Carotenogenesis in fruits also triggers an increase in the number and size of plastoglobules and membranous structures needed for carotenoid storage (Harris and Spur, 1969). Plastoglobules are composed of lipids and several proteins, of which plastoglobulin (also called plastid lipid-associated protein, PAP3) and chromoplast-specific carotenoid-associated protein (CHRC) are believed to assist in the storage of carotenoids (Kessler *et al.*, 1999; Kilambi *et al.*, 2013; Nogueira *et al.*, 2013). It is also reported that plastoglobules harbor enzymes that regulate carotenoid metabolism such as phytoene synthase (PSY), ζ-carotene desaturase (ZDS), lycopene β-cyclase, and β-carotene hydroxylases (Ytterberg *et al.*, 2006; Shumskaya *et al.*, 2012).

Currently, regulation of fruit coloration in the tomato clade is incompletely understood. It is presumed that the ‘green-fruitedness’ in the tomato clade is due to under-expression of key carotenogenic genes like *psy1* (Meléndez-Martínez *et al.*, 2010; Bolger *et al.*, 2014). Recent transcriptome analysis revealed that the absence of coloration in green-fruited *S. pennellii* may have stemmed from the dominant expression of photosynthesis-related genes and the absence of *phytoene synthase 1* expression (Bolger *et al.*, 2014). Compared with carotenogenic gene expression, little is known about the proteome complement involved in carotenoid biosynthesis and sequestration in the tomato clade.

To comprehend the diversity of fruit coloration, we combined carotenoid profiles and carotenogenic gene expression with the proteome complement to decipher likely metabolic and structural blocks restricting fruit coloration in wild species of the tomato clade. Comparison of the green-fruited *S. habrochaites* (SH), orange-fruited *S. galapagense* (SG), and red-fruited *S. pimpinellifolium* (SP) with red-fruited domesticated *S. lycopersicum* (SL) revealed wide diversity in profiles of gene expression, proteome complement, and carotenoid levels. We show that the green-fruited SH, though it encoded a functional PSY1, lacked fruit-specific carotenogenesis owing to multiple blocks spanning several facets of carotenoid accumulation, carotenoid sequestration and chromoplast formation.

## Materials and methods

### Plant material

The tomato *Solanum lycopersicum* cv. Ailsa Craig (LA2838) and its wild relatives *S. pimpinellifolium* (LA1589), *S. galapagense* (LA0483), and *S. habrochaites* (LA1777) were used in this study. Plants were grown in an open field in pots as per the experimental design (see Supplementary Fig. S1 at *JXB* online) during October–January at the University of Hyderabad (day, 30 ± 2 °C; night, 18 ± 2 °C). The flowers were tagged on the day of anthesis, fruit growth was periodically monitored, and fruits were collected at 26, 29 and 39 d post-anthesis (dpa) (Supplementary Fig. S2). The pericarps were collected from fruits, snap frozen in liquid nitrogen, and stored at −80 °C until use. The ripe fruits were also collected from *S. pennellii* introgression lines IL3-2 and IL6-3 and stored at −80 °C.

### Statistical analysis

Unless otherwise mentioned, a minimum of three biological replicates was used for every experiment. Based on the samples, either Student’s *t*-test or one-way ANOVA followed by the Holm–Sidak test was performed to determine significant differences using Sigmaplot (version 11.0). Where required, the *P* values <0.05, <0.01, <0.001, <0.0001 are indicated by one, two, three, or four asterisks, respectively.

### Determination of ethylene, carotenoids, and transcript levels

Ethylene emission was measured as described by Kilambi *et al.* (2013). The carotenoid and chlorophyll contents were analysed as per the protocols of Gupta *et al.* (2015) and Wellburn (1994), respectively. Total RNA was isolated using TRI reagent (Sigma-Aldrich) and q-PCR was carried out as described earlier (Kilambi *et al.*, 2013) (for primer details, see Supplementary Table S1A).

### Transmission electron microscopy

Fruits collected at 39 dpa were sliced into 2 mm pieces and fixed overnight in 2.5% (v/v) glutaraldehyde in 0.1 M sodium phosphate buffer, pH 7.2 at 4 °C and thereafter processed as described in Saladié *et al.* (2007). Sections were viewed with a JEM-2100F transmission electron microscope (JEOL, Peabody, MA, USA).

### Total protein extract preparation and shotgun proteomics approach

Proteins from fruit tissue were extracted as described in Kilambi *et al.* (2013). For proteome profiling, a corresponding sample from SL was processed in parallel with the respective wild species from the protein extraction to liquid chromatography–mass spectrometry (LC-MS) separation. Protein (70 µg) from each genotype and stage was subjected to one dimensional SDS-PAGE followed by LC-MS (GeLC-MS) as described in Kilambi *et al.* (2016) and illustrated in Supplementary Fig. S3. For SH–SL samples, protein was solubilized in lysis buffer containing 2% (w/v) 3-(4-heptyl) phenyl-3-hydroxypropyl dimethyl ammoniopropane sulfonate (C7BzO) and 2% (w/v) CHAPS prior to GeLC-MS. In-gel digestion was carried out and peptides were extracted following the protocol of Kilambi *et al.* (2016). Peptides were desalted through C18 spin columns (Thermo Scientific) and were aliquoted and dried, and stored at −80 °C.

### Mass spectrometry and data analysis

The trypsin digests were analysed using an Easy-nLC II coupled to an LTQ Orbitrap-VelosPro mass spectrometer (Thermo Scientific). The gradients and mass spectrometry parameters used were described earlier (Kilambi *et al.*, 2016) and for SH–SL comparison, a shorter run time of 90 min was used. Data were analysed using Proteome Discoverer version 1.4 (Thermo Scientific). The databases (for SL) and search parameters (for all genotypes) used were described in Kilambi *et al.* (2016). For SP and SG, data were searched against the *S. pimpinellifolium* database (ftp://ftp.solgenomics.net/genomes/Solanum_pimpinellifolium/annotations/Spimpinellifolium _genome_protein_annot.fa) containing 32715 sequences, 11 112 863 residues, downloaded on 25 April 2013). For SH, data were searched against protein database generated from SH RNA-Seq data obtained from Julin Maloof’s lab (14369 sequences, 6 321 576 residues, downloaded on 26 March 2015). Label-free quantification based on signal intensity (Sieve 2.1 software, Thermo Scientific) was used to identify the differentially expressed proteins in SH–SL, SP–SL and SG–SL pairs after passing stringent selection criteria (*P* value≤0.05, minimum 2-fold change (either up- or down-regulation), minimum two peptides for identification, FDR=1%). The mass spectrometry proteomics data have been deposited to the ProteomeXchange Consortium (Vizcaíno *et al.*, 2014) via the PRIDE partner repository with the dataset identifier PXD003817.

### Targeted peptide monitoring for carotenoid pathway enzymes

Peptides pertaining to the carotenogenic proteins at 39 dpa from the proteome data were chosen for targeted peptide monitoring (TPM) as described in Lange *et al.* (2008) and Taylor *et al.* (2014). A list of proteotypic peptides was generated manually for SL, SP, SG, and SH. The number of peptides chosen for TPM was 96 for SH–SL, 97 for SP–SL and 116 for SG–SL comparisons (see Supplementary Table S2). For TPM, data-independent settings were used as described in Supplementary Fig. S4. The carotenogenic peptides were quantified in a label-free manner using internal control peptides (Supplementary Table S3) as described in Supplementary Fig. S4.

### Western blot analysis

Proteins extracted from 39 dpa fruits of SL, SP, SG, and SH, were separated on SDS- PAGE followed by western blot analysis using a previously described protocol (Kilambi *et al.*, 2013). For western blot analysis of CHRC and PAP3, polyclonal antibodies against bell pepper PAP (1/2500; Kilambi *et al.*, 2013) and plastoglobulin (Agrisera AS06 116, 1/1000), respectively, were used.

### Photosystem II, and pheophorbide *a* oxygenase/red chlorophyll catabolite reductase activity

The activity of PSII was determined using the Hill reaction (Bregman, 1990). The pheophorbide *a* oxygenase (PAO) and red chlorophyll catabolite reductase (RCCR) enrichment from fruit tissue and PAO and RCCR enzyme assays were carried out as described earlier (Hörtensteiner *et al.*, 1995, Christ *et al.*, 2012).

### Functional expression of *psy1* and *cycb* genes in *E. coli*

pAC-85b and pAC-LYC were gifts from Francis X. Cunningham Jr (Addgene plasmid nos 53282 and 53270). *E. coli* TOP 10 competent cells carrying pAC-85b were co-transformed with pET32a-*psy1*-SL, pET32a-*psy1*-SH and pET32a-*psy1*-IL3-2 constructs (Cunningham and Gantt, 2007), while the cells carrying pAC-LYC were co-transformed with pET32a-*cycb*-SL, pET32a-*cycb*-SH, and pET32a-*cycb*-IL6-3 constructs (Cunningham *et al.*, 1994) (for primer details, see Supplementary Table S1B). Transformed cells were selected on LB agar plates, grown in 50 mL LB medium supplemented with 37 mg ml^−1^ chloramphenicol and 50 mg ml^−1^ kanamycin at 37 °C. The bacterial cultures were harvested by centrifugation at 4000 *g* for 10 min, and total carotenoids were analysed as described in Gupta *et al.* (2015).

## Results

### Carotenoid profiling

We compared the fruits of SH, SG, and SP with reference to SL ripening stages, i.e. the green stage at 26 d post-anthesis (dpa), the transitional stage at 29 dpa and the orange/red stage at 39 dpa (26→39 dpa) (see Supplementary Fig. S2). Though SH fruits lack color transitions, we used the above time points for SH too. In orange/red-fruited species, increase in carotenoid levels (26→39 dpa) was associated with a decline in chlorophyll levels. However, SH fruits retained a similar level of carotenoids and chlorophylls (Table 1). The initiation of ripening led to a preferential accumulation of lycopene/β-carotene and appearance of phytoene and phytofluene at 39 dpa in all species, except SH. In addition, lycopene/β-carotene ratios revealed a shift towards higher lycopene levels (SH<SG<SL<SP). Examination of the ratios between the β-carotene and α-carotene pathways revealed that the β-carotene pathway was predominant in SP at 26 dpa (SP>SL>SG>SH) and in SG at 39 dpa (SG>SL>SP>SH). The chlorophyll (Chl)/carotenoid (Car) ratio in fruits showed a continual decline (26→39 dpa) except in SH.

**Table 1.** Carotenoid and chlorophyll contents in the fruits of wild species and tomato Carotenoid and chlorophyll contents are presented as μg/g fresh weight. Data are means±SE (*n*=3). Chl, chlorophyll; Car, carotenoid; ND, not detected as no peak was discernible on the chromatogram. The Holm–Sidak test was used to determine the significant differences in the metabolites between the wild species and tomato. Values in bold indicate significant differences: *P<0.05, **P<0.01 and ***P<0.001.

Metabolite (µg gFW^−1^)	*S. lycopersicum*			*S. pimpinellifolium*			*S. galapagense*			*S. habrochaites*		
	26 dpa	29 dpa	39 dpa	26 dpa	29 dpa	39 dpa	26 dpa	29 dpa	39 dpa	26 dpa	29 dpa	39 dpa
Phytoene	ND	ND	13.16 ± 0.97	ND	ND	66.86 ± 2.31***	ND	ND	34.35 ± 1.2***	ND	ND	ND
Phytofluene	ND	ND	2.47 ± 0.12	ND	ND	7.08 ± 0.50***	ND	ND	2.55 ± 0.23	ND	ND	ND
Lycopene	ND	ND	64.59 ± 9.3	ND	ND	189.24 ± 11.31***	ND	0.29 ± 0.004***	1.54 ± 0.05***	ND	ND	ND
α-Carotene	ND	ND	ND	ND	ND	ND	ND	ND	3.11 ± 0.48***	ND	ND	ND
β-Carotene	ND	3.85 ± 0.17	12.31 ± 0.3	2.08 ± 0.30	2.36 ± 0.28	8.30 ± 0.50	3.96 ± 0.54***	5.27 ± 1.04	30.13 ± 3.13**	3.76 ± 0.08***	2.75 ± 0.17	4.42 ± 0.27**
Lutein	ND	3.56 ± 0.21	4.62 ± 0.6	3.82 ± 0.33	3.75 ± 0.61	5.08 ± 0.13	7.46 ± 0.88***	6.41 ± 0.96**	5.07 ± 0.48	6.57 ± 0.52***	3.88 ± 0.26	5.36 ± 0.40
Zeaxanthin	ND	ND	ND	ND	ND	ND	1.22 ± 0.17***	1.13 ± 0.21***	ND	ND	ND	ND
Violaxanthin	1.64 ± 0.12	2.49 ± 0.29	1.19 ± 0.01	3.0 ± 0.18***	1.82 ± 0.12	1.22 ± 0.03	3.16 ± 0.36***	3.37 ± 0.57	1.73 ± 0.14*	2.08 ± 0.12	2.47 ± 0.23	2.22 ± 0.2***
Antheraxanthin	1.09 ± 0.01	2.13 ± 0.06	ND	1.43 ± 0.04***	2.26 ± 0.08	ND	ND	2.48 ± 0.27	ND	1.21 ± 0.02**	2.19 ± 0.18	ND
Neoxanthin	1.12 ± 0.02	0.90 ± 0.08	ND	1.64 ± 0.05**	1.23 ± 0.07	ND	1.73 ± 0.21**	2.83 ± 0.51***	ND	1.01 ± 0.07	1.33 ± 0.027	0.99 ± 0.12***
Total Car	9.16 ± 0.37	12.93 ± 0.52	101.62 ± 8.5	11.97 ± 0.44**	11.42 ± 0.41	277.78 ± 25.3***	17.53 ± 1.04***	21.78 ± 0.7	78.48 ± 5.4***	14.63 ± 1.03***	12.62 ± 0.41	12.99 ± 0.99***
Chlorophyll *a*	73.25 ± 1.27	44.9 ± 0.93	24.6 ± 0.81	108.5 ± 5.7***	57.5 ± 0.5***	23.32 ± 0.19*	124.47 ± 13.2***	97.7 ± 3.8***	25.4 ± 0.31*	74.75 ± 2.4*	65.9 ± 1.3***	67.74 ± 5.04***
Chlorophyll *b*	46.1 ± 0.61	43.2 ± 1.42	38.28 ± 0.87	59.8 ± 0.52***	44.2 ± 1.25*	36.8 ± 0.59*	69.5 ± 4.4***	59.9 ± 5.3***	38.45 ± 0.67*	49.7 ± 1.08*	46 ± 0.23*	49.23 ± 0.87***
Chl *a*: Chl *b*	1.59 ± 0.009	1.04 ± 0.09	0.64 ± 0.006	1.81 ± 0.06*	1.3 ± 0.025***	0.63 ± 0.01	1.79 ± 0.07*	1.64 ± 0.08***	0.66 ± 0.005	1.5 ± 0.02*	1.43 ± 0.02***	1.37 ± 0.04***
Total Chl	119.35 ± 0.1	88.1 ± 2.54	62.88 ± 1.25	168.3 ± 2.34*	101.7 ± 0.75**	60.12 ± 0.6*	193.97 ± 10.22*	157.6 ± 5.4***	63.85 ± 0.75	124.45 ± 2*	111.9 ± 0.7***	116.97 ± 3.5***
Chl:Car	13.2 ± 8.45	6.81 ± 0.073	0.61 ± 0.032	14 ± 0.654***	8.9 ± 0.76**	0.216 ± 0.019***	11.06 ± 0.12	7.23 ± 0.79**	0.81 ± 0.08***	8.5 ± 0.83***	8.86 ± 0.9***	9.00 ± 0.79***

### Ethylene emission is not coupled to carotenoid formation in tomato’s wild relatives

**Fig. 1.**
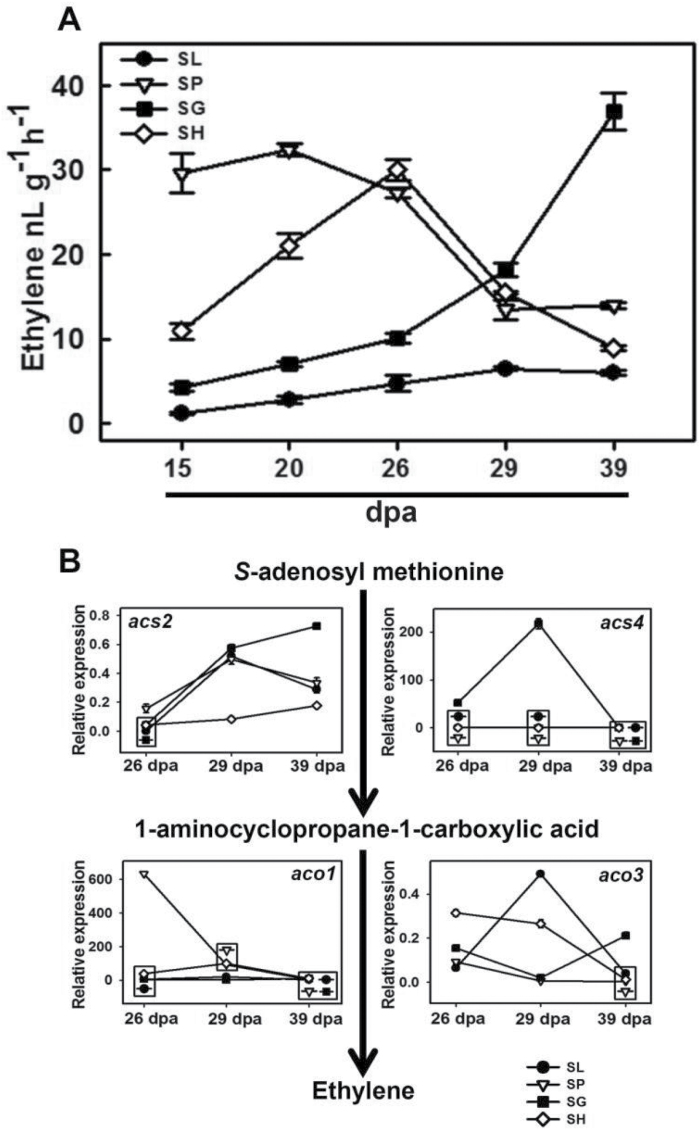
Ethylene emission and transcript levels of ethylene biosynthesis genes in tomato and wild species. (A) Fruits were harvested at the indicated time points, and ethylene emission was measured. Data are means±SE (*n*=5), *P*≤0.05 and presented in Table S7). (B) The relative transcript levels of *acs2*, *acs4*, *aco1*, and *aco3* in 26, 29, and 39 dpa fruits were measured after normalization with *β-actin* and *ubiquitin* (Table S7 shows means±SE (*n*=3) along with corresponding *P*-values). The overlapping symbols are enclosed in a box. Error bars smaller than the symbol are masked underneath.

Considering the integral role of ethylene in tomato fruit ripening and carotenoid accumulation, we examined ethylene emission in the wild species. Measurement of ethylene emission from the fruits of SL and its wild relatives at 15→39 dpa, revealed that all wild relatives emitted more ethylene than SL at all stages (Fig. 1A and Supplementary Fig. S2). However, unlike SL, ethylene emission in wild relatives was not correlated with carotenoid accumulation. Even in red-fruited SP, the carotenoid accumulation did not coincide with the ethylene burst. In SL, ethylene emission is reportedly preceded by up-regulation of *1-aminocyclopropane-1-carboxylate synthase 2* and *4* (*acs2*, *acs4*), *1-aminocyclopropane-1-carboxylic acid oxidase 1* and *3* (*aco1*, *aco3*) transcripts (Barry *et al.*, 2000). In each wild species, the expression profiles and the relative levels of these transcripts followed distinctly different patterns (Fig. 1B).

### Expression profiles of carotenogenic genes and ripening regulators

**Fig. 2.**
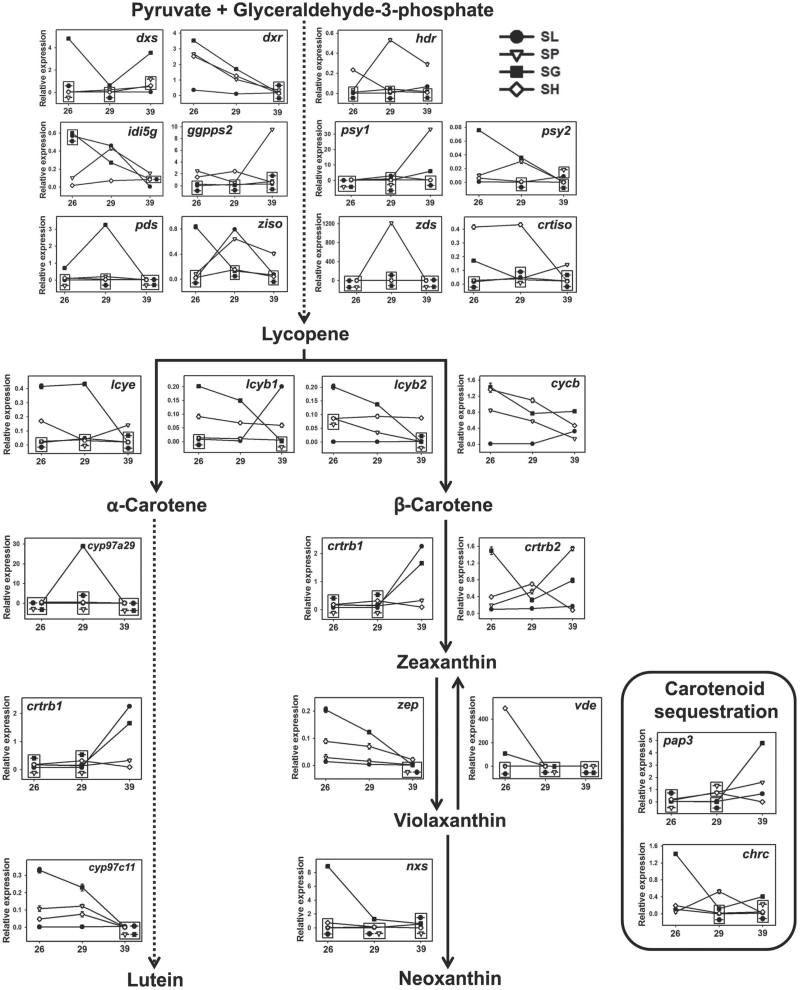
Relative expression of genes mediating carotenoid biosynthesis and sequestration in tomato and wild species. The relative transcript levels were measured from 26, 29 and 39 dpa fruits after normalization with *β-actin* and *ubiquitin* (Table S7 shows means±SE (*n*=3) along with corresponding *P*-values). Dotted arrows indicate multiple steps in the carotenoid biosynthesis pathway. The genes mediating carotenoid sequestration are enclosed in a rectangle. The overlapping symbols are enclosed in a box. Error bars smaller than the symbol are masked underneath. *chrc*, *chromoplast-specific carotenoid associated protein*; *crtiso*, *carotenoid isomerase*; *crtrb1*, *β-carotene hydroxylase 1*; *crtrb2*, *β-carotene hydroxylase 2*; *cycb*, *chromoplast-specific lycopene β-cyclase*; *cyp97a29*, *cytochrome P450 carotenoid β-hydroxylase A29*; *cyp97c11*, *cytochrome P450 carotenoid ε-hydroxylase C11*; *dxr*, *deoxy-xylulose 5-phosphate reductase*; *dxs*, *deoxy-xylulose 5-phosphate synthase*; *ggpps2*, *geranylgeranyl diphosphate synthase 2*; *hdr*, *4-hydroxy-3-methylbut-2-enyl diphosphate reductase*; *idi5g*, *isopentenyl diphosphate isomerase 5g*; *lcyb1*, *lycopene β-cyclase 1*; *lcyb2*, *lycopene β-cyclase 2*; *lcye*, *lycopene *ε*-cyclase*; *pds*, *phytoene desaturase*; *nxs*, *neoxanthin synthase*; *pap3*, *plastid lipid-associated protein 3*; *psy1*, *phytoene synthase 1*; *psy2*, *phytoene synthase 2*; *vde*, *violoxanthin deepoxidase*; *zds*, *ζ-carotene desaturase*; *zep*, *zeaxanthin epoxidase*; *ziso*, *ζ-carotene isomerase*.

The geranylgeranyl diphosphate (GGPP) required for phytoene formation is derived from the plastid-localized methylerythritol-4-phosphate (MEP) pathway. The genes *deoxy-xylulose 5-phosphate synthase* (*dxs*), *deoxy-xylulose 5-phosphate reductase* (*dxr*), *4-hydroxy-3-methylbut-2-enyl diphosphate reductase* (*hdr*), *isopentenyl diphosphate isomerase 5g* (*idi5g*) and *ggpp synthase 2* (*ggpps2*) leading to GGPP formation were up-regulated in all wild species compared with SL barring a few exceptions (Fig. 2). Relative to SL, only a few genes were down-regulated (*dxs*, 26 dpa SP, 29 dpa SH; *hdr*, 39 dpa SG, SH; *ggpps2*, 29 dpa SG; *idi5g*, 26 dpa SP, SH; 29 dpa SH, SG).

In tomato, PSY1 and PSY2 reportedly contribute to fruit- and leaf-specific carotenoid formation, respectively. Consistent with this, *psy1* expression in orange/red fruits was substantially higher than *psy2* with high levels in SP and SG at 39 dpa. Though SH is green fruited, it showed a high level of *psy1* compared with *psy2* (66-, 158-, and 860-fold higher at 26, 29 and 39 dpa, respectively). The conversion of phytoene to lycopene is contributed by four genes, *phytoene desaturase* (*pds*), *ζ-carotene isomerase* (*ziso*), *zds* and *carotenoid isomerase* (*crtiso*). Out of three *lycopene β-cyclases* [*lycopene β-cyclase 1* (*lcyb1*), *lycopene β-cyclase 2* (*lcyb2*) and *chromoplast-specific lycopene β-cyclase* (*cycb*)] in tomato, only *cycb* drives the β-carotene formation in fruits. Consistent with this, *cycb* expression in fruits was higher than *lcyb1* and *lcyb2*; its expression declined in the wild species while it increased in SL (26→39 dpa). Though SH formed little β-carotene, it had higher *cycb* expression than orange/red-fruited species. The expression profile of *lcyb1* was similar to *cycb*, whereas *lcyb2* expression gradually declined in SP and SG and remained constant in SL and SH. The expression profiles of genes downstream to β-carotene were different in each species.

In conjunction with carotenoid biosynthetic genes, the expression of sequestration genes, i.e. *chrc* and *pap3* was also examined. The orange/red-fruited species showed high expression of *pap3* at 39 dpa, whereas in SH, it peaked at 29 dpa. The *chrc* expression in SL, SG, and SH declined at 29 dpa and increased after that, while SP showed a peak at 29 dpa.

We further examined whether the green-fruited nature of SH resulted from the lack of or subdued expression of transcription factors regulating fruit ripening. In general, the expression levels of most transcription factors were higher in wild species, including SH, than SL, the exception being *ethylene response factor 6* (*erf6*) and *HD-Zip homeobox protein1* (*hb1*) (39 dpa) (see Supplementary Fig. S5). The expression of *colorless nonripening* (*cnr*) increased in red/orange-fruited species, whereas in SH it declined (26→39 dpa).

### Fruit proteome analysis

**Fig. 3.**
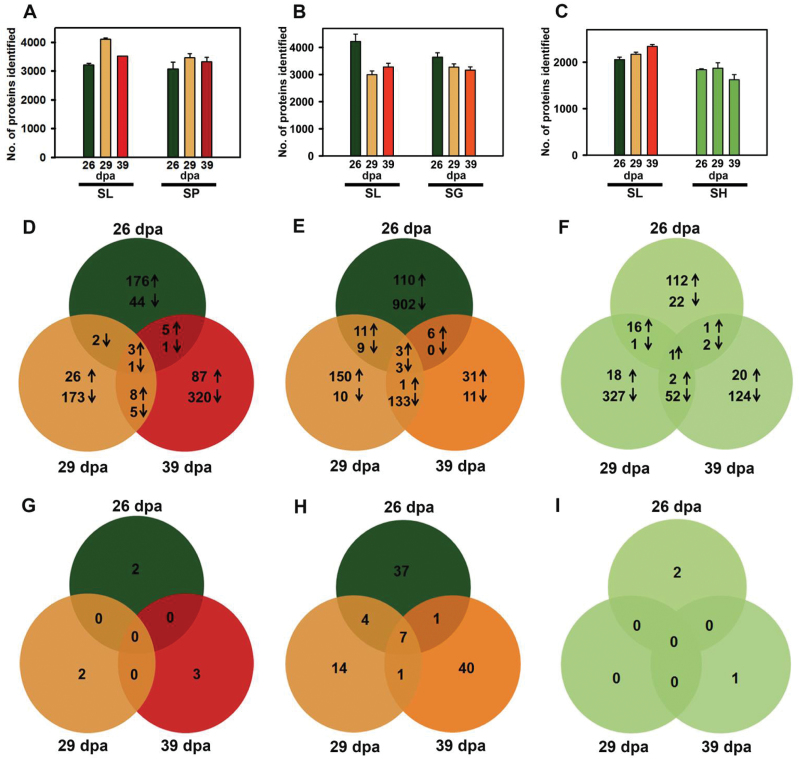
Proteome analyses from the fruits of tomato and wild species. The fruits were harvested at 26, 29, and 39 dpa. Data are means±SE (*n*=3), *P*≤0.05; up-regulation (↑), down-regulation (↓)]. (A–C), Number of proteins identified in fruits. (A) SP and SL; (B) SG and SL; (C) SH and SL. (D–F) Differentially expressed proteins in wild species in comparison with SL and listed in Table S4. (D) SP; (E) SG; (F) SH. (G–I) Proteins identified solely in wild species and listed in Table S5. (G) SP; (H) SG; (I) SH. (This figure is available in color at *JXB* online.)

Using a shotgun proteomics approach, a wide spectrum consisting of 3250–3500 proteins was detected in orange/red-fruited species (Fig. 3A, B and Supplementary Fig. S3). In SH, a slimy component interfered with protein extraction necessitating modification of the protocol. Though protein yield was reduced, an average of 2188 and 1777 proteins were identified in SL and SH fruits, respectively (Fig. 3C). Comparison of the wild species’ proteomes with SL revealed a large number of differentially expressed proteins in wild relatives (SP, 851; SG, 1440; SH, 698) (Fig. 3D–F and Supplementary Table S4). On comparing the overall proteome profile with SL, few proteins were detected only in the wild relatives (SP, 7; SG, 104; SH, 3) at different stages (Fig. 3G–I and Supplementary Table S5). In SL, no peptide matches corresponding to these proteins were found at any stage and most of these proteins constituted different isoforms of existing proteins (see Supplementary Tables S4 and S5).

### Functional classification of differentially expressed proteins

The differentially up-regulated (SP, 41; SG, 42; SH, 41) or down-regulated (SP, 42; SG, 44; SH, 41) proteins compared with SL were classified using MapMan (Usadel *et al.*, 2009; Supplementary Fig. S6 and Supplementary Table S4). The major up-regulated class varied in all wild relatives: in SP it was development and storage (16.7%), in SG it was miscellaneous (7.6%) followed by protein degradation (7.3%), whereas in SH it was protein synthesis (9.3%). Consistent with chlorophyll retention in SH fruits, the functional classes PS-light reaction and Calvin cycle were highly up-regulated (9.2%) in SH, whereas these were up-regulated by 6.1% in SG and 5.8% in SP (see Supplementary Fig. S5). The functional class protein degradation in SP (6.2%) and SG (7.3%) was strongly up-regulated; conversely, SH showed up-regulation of protein synthesis (9.3%). Ostensibly fruit development in SH follows a different trajectory from SG and SP. Similarly, among the down-regulated proteins, variation was observed among the wild relatives. In SP, the functional class abiotic stress (13.8%) was the major down-regulated category; in SG, it was protein synthesis (15.2%), while it was protein degradation (9.9%) in SH.

### Protein functional classes involved in chromoplast formation

**Fig. 4.**
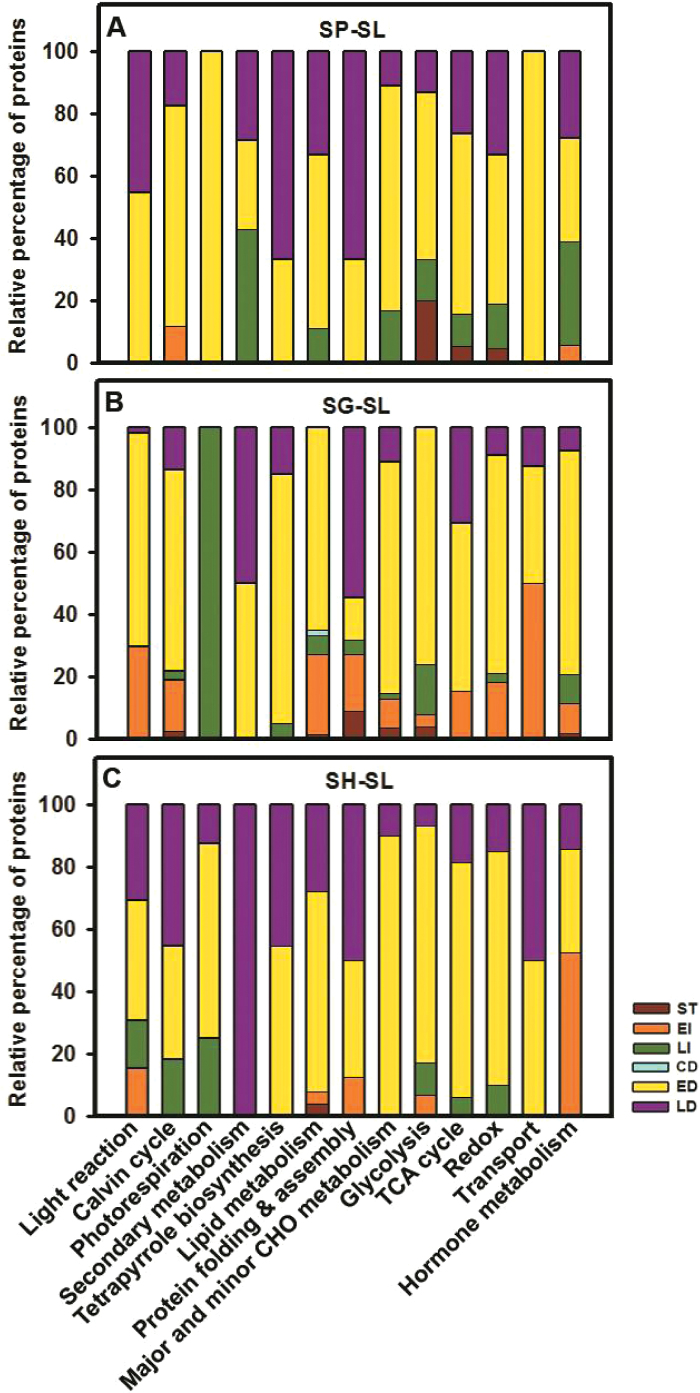
Abundance patterns of proteins involved in chromoplast formation. Proteins belonging to the functional classes with a presumed role in chromoplast formation from SP–SL (A), SG–SL (B) and SH–SL (C) were differentiated based on their abundance pattern as stable (ST), continuous increase (CI), early increase (EI), late increase (LI), continuous decrease (CD), early decrease (ED), and late decrease (LD), and presented in Table S6. The individual bars represent different protein functional categories. The relative percentage of proteins exhibiting the above abundance patterns is represented in the respective bars. (This figure is available in color at *JXB* online.)

During chromoplast formation, the plastid ultrastructure is modified to store carotenoids in membranous structures and plastoglobules. This storage is facilitated by up-regulation of carotenoid sequestration proteins like CHRC (Kilambi *et al.*, 2013) and PAP3 (Kessler *et al.*, 1999; Nogueira *et al.*, 2013). The transition to the chromoplast also involves wide alterations in the proteome (Barsan *et al.*, 2012). The differentially expressed proteins involved in chromoplast formation (Barsan *et al.*, 2012) were classified based on their abundance patterns into seven categories: stable, continuous increase, early increase, late increase, continuous decrease, early decrease, and late decrease (Fig. 4 and Supplementary Table S6). Overall, a majority of the proteins decreased in abundance across all the genotypes (SP–SL: 0 continuous + 83 early + 42 late=125; SG–SL: 1 continuous + 256 early + 40 late=297 and SH–SL: 0 continuous + 114 early + 43 late=157).

Comparison of proteome profiles of different wild relatives revealed that though most proteins involved in chromoplast formation behaved similarly to tomato, several species-specific differences were also discernible (see Supplementary Table S6). For SP–SL, SG–SL, and SH–SL, proteins belonging to photosynthesis, Calvin cycle, photorespiration, lipid metabolism, protein assembly and folding, major and minor CHO metabolism, tetrapyrrole biosynthesis, transport, glycolysis, redox, TCA cycle, and hormone metabolism categories decreased in abundance. The proteins (20%) in the glycolysis category in SP–SL remained stable, while proteins (9%) in the protein assembly and folding category were stable in SG–SL. Unlike other wild species, proteins (52%) in the hormone metabolism category increased in abundance in SH.

### SH fruits retain chloroplasts and functional photosystem II

**Fig. 5.**
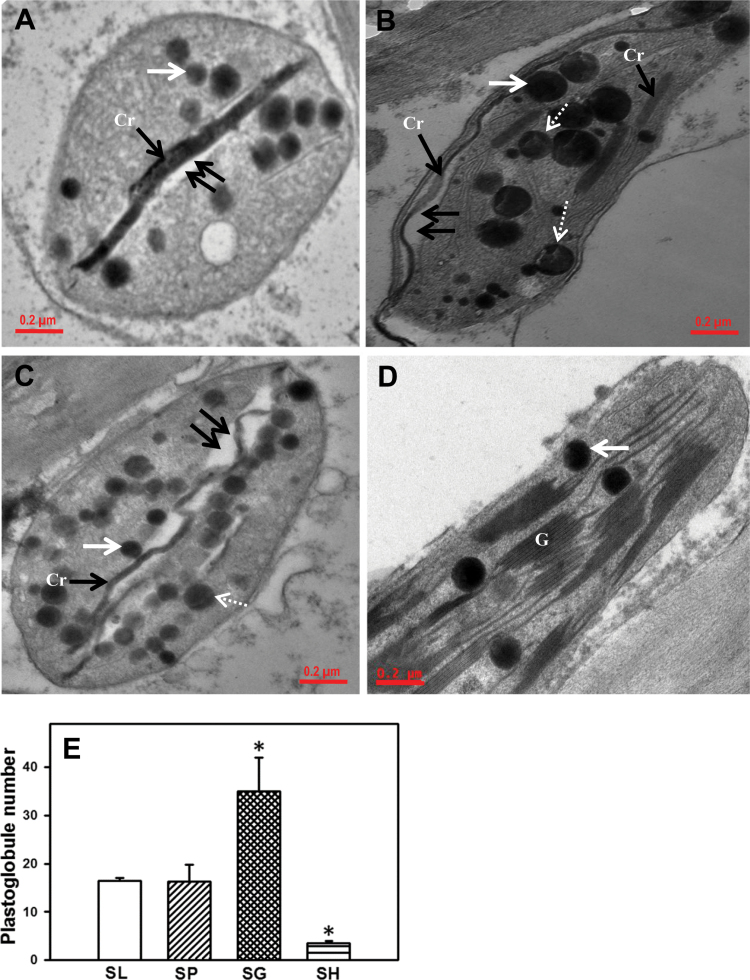
Transmission electron micrographs of chromoplasts/chloroplasts present in the 39 dpa fruits of tomato and wild relatives. (A) SL; (B) SP; (C) SG; (D) SH. Double arrows show an enlarged granal compartment, black arrows show lycopene crystalloid, white arrows indicate plastoglobules, and dashed white arrows show morphologically distinct plastoglobules; Cr, lycopene crystal; G, granum. (E) Relative number of plastoglobules present in chloroplast/chromoplasts of tomato and wild species. Data are means±SE (*n*=3), *P≤0.05.

**Fig. 6.**
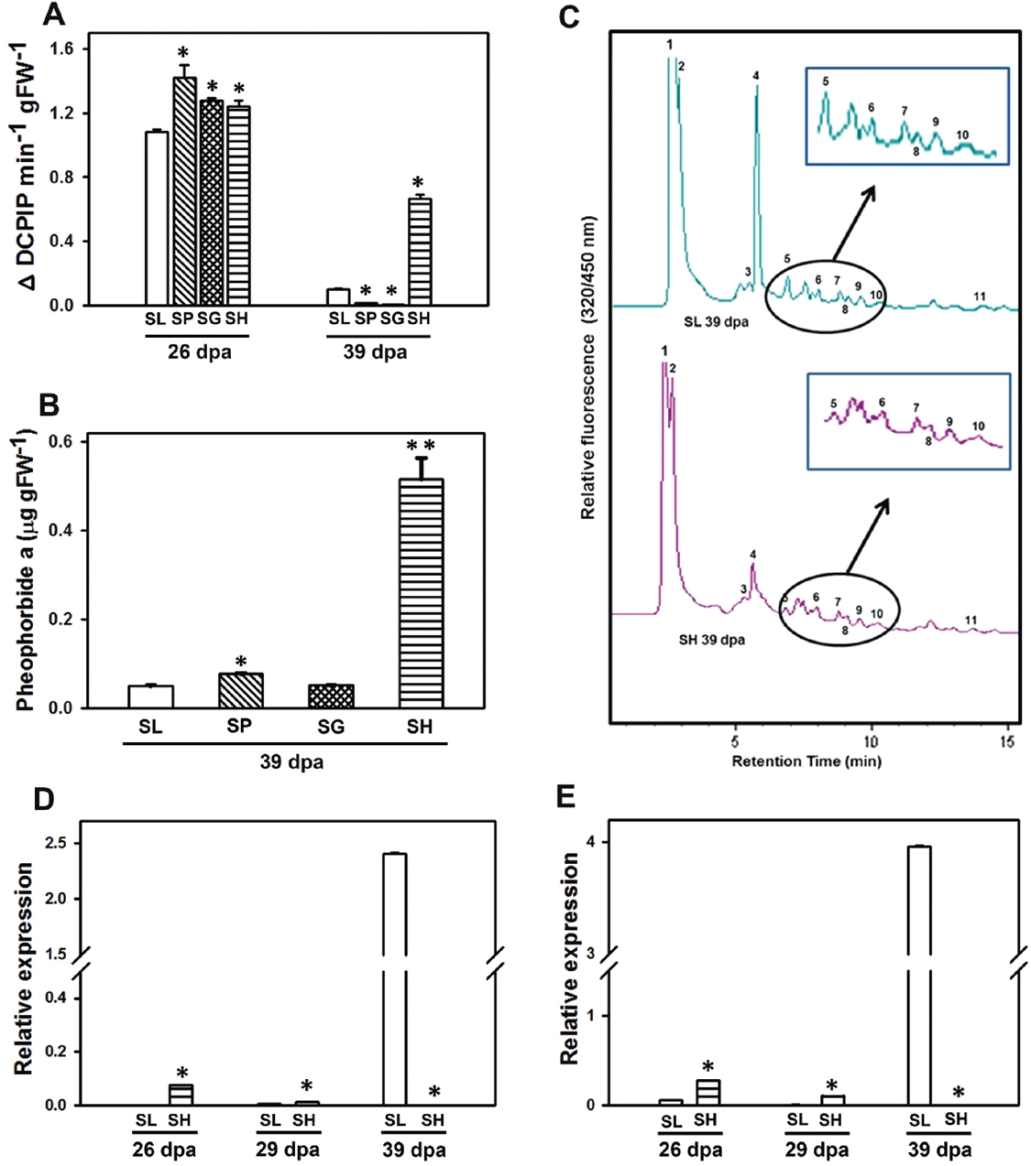
Photosynthesis and chlorophyll degradation. (A) Photosystem II activity in 26 and 39 dpa fruits of tomato and wild species. (B) Pheophorbide *a* levels in 39 dpa fruits of tomato and wild species. (C) HPLC traces of PAO/RCCR assays using 39 dpa fruit extracts of SL and SH. The inset shows enlarged view of the peaks. The peak areas are presented in Supplementary Fig. S7. (D, E) Relative transcript levels of *pao* (D) and *rccr* (E). Relative transcript expression levels were obtained after normalization with *β-actin* and *ubiquitin*. Data are means±SE (*n*=3), *P≤ .05, **P≤0.01. Supplementary Table S7 shows means±SE (*n*=3) along with corresponding *P*-values for relative transcript expression of *pao* and *rccr*. (This figure is available in color at *JXB* online.)

During fruit development (26→39 dpa), chlorophyll levels and the Chl/Car ratio declined in SL, SP and SG, while SH fruits sustained these at a high level (Table 1). This was also reflected in the ultrastructure of the plastids. The chromoplasts of 39 dpa SL, SP and SG fruits showed reduced granal membranes and increased number of plastoglobules (Fig. 5A–C). The green-fruited SH retained chloroplasts with intact thylakoid membranes with fewer plastoglobules (Fig. 5D). Consistent with high carotenoid levels, SP had large plastoglobules, whereas in SG plastoglobules were many and smaller than those of SL (Fig. 5E). Lycopene crystalloids were predominant in red-fruited SP and crystalloid remnants of lycopene associated with undulating internal membranes were observed in SL, SP, and SG, but not in SH. The retention of a high Chl/Car ratio indicated sustenance of photosynthetic activity in SH fruits as evident by the PSII-mediated evolution of oxygen. While fruits of all genotypes showed high PSII activity at 26 dpa, it massively declined in red/orange-fruited species (SL 11%; SP 1.1%; SG 0.5%) at 39 dpa, whereas SH fruits retained >50% of PSII activity (Fig. 6A).

### SH fruits show a block in the pheophorbide *a*/phyllobilin pathway

The SH fruit also showed reduced levels of chlorophyll destabilization (Fig. 6). This was corroborated by the persistence of chlorophyll-*a*/*b* binding protein (3-fold), oxygen evolving enhancer protein 2 (8.6-fold), Rubisco activase 1 (4-fold) and fructose bisphosphate aldolase (3.5-fold) in SH fruits at 39 dpa (see Supplementary Table S4), while these proteins declined in SP and SG. In higher plants, chlorophyll degradation is mediated by the pheophorbide *a* (PAO)/phyllobilin pathway (Christ and Hörtensteiner, 2014). The 10.5-fold higher pheophorbide *a* level in SH fruits at 39 dpa than in SL (Fig. 6B) indicated that chlorophyll catabolism is not fully functional in SH. Among eight proteins belonging to the PAO pathway, red chlorophyll catabolite reductase (RCCR) and PAO were detected in SP and SG but not in SH fruits. The coupled PAO–RCCR assay in 39 dpa fruits supported lower PAO/RCCR activity in SH than SL (Fig. 6C). Consistent with lower PAO/RCCR activity, SH fruits also showed low levels of primary fluorescent catabolites (Supplementary Fig. S7) as well as low expression of *pao* and *rccr* genes than SL (Fig. 6D, E).

### Targeted peptide monitoring of carotenogenic proteins in wild relatives

In higher plants, enzymes of the carotenoid biosynthesis pathway are present at very low levels and are highly labile to purify (Hirschberg, 2001). Examination of proteome profiles in this study detected about 20 proteins related to carotenogenesis, sequestration, and cleavage. We could not detect β-carotene hydroxylases, CYP97A11, and CYP97A29, perhaps due to low abundance as even in isolated tomato chromoplasts these proteins were not detected (Barsan *et al.*, 2012; Wang *et al.*, 2013). Among the differentially expressed proteins, few proteins related to carotenoid metabolism and accumulation showed both up-regulation (SP: CHRC, 9.17-fold; carotenoid cleavage dioxygenase 1B (CCD1B), 6.21-fold; SG: isopentenyl diphosphate isomerase 5G (IDI5G), 13.5-fold) and down-regulation (SH: geranyl geranyl diphosphate synthase isoform 9G (GGPPS9G), 25-fold; carotenoid isomerase (CRTISO), 72-fold; lycopene β-cyclase 1 (LCYB1), 63-fold) (see Supplementary Table S4).

To establish a linkage between carotenoids and carotenogenic proteins, a targeted peptide monitoring approach (Hewel *et al.*, 2013; see Supplementary Fig. S4) was used as it can quantify very low abundance proteins including isoforms (Lehmann *et al.*, 2008; Aebersold *et al.*, 2013; Taylor *et al.*, 2014). We used label-free quantification for targeted peptide monitoring (TPM) by using internal proteins as controls (Supplementary Fig. S4 and Supplementary Table S3) (Sherrod *et al.*, 2012; Carr *et al.*, 2014). In consonance with high carotenoid levels, a maximum number of proteins mediating carotenoid accumulation were detected at 39 dpa. In view of this, we selected 39 dpa as the time point for TPM. Drawing from proteome profiles, around 20 proteins contributing to carotenogenesis, sequestration, and cleavage were selected for TPM (see Supplementary Table S2).

**Fig. 7.**
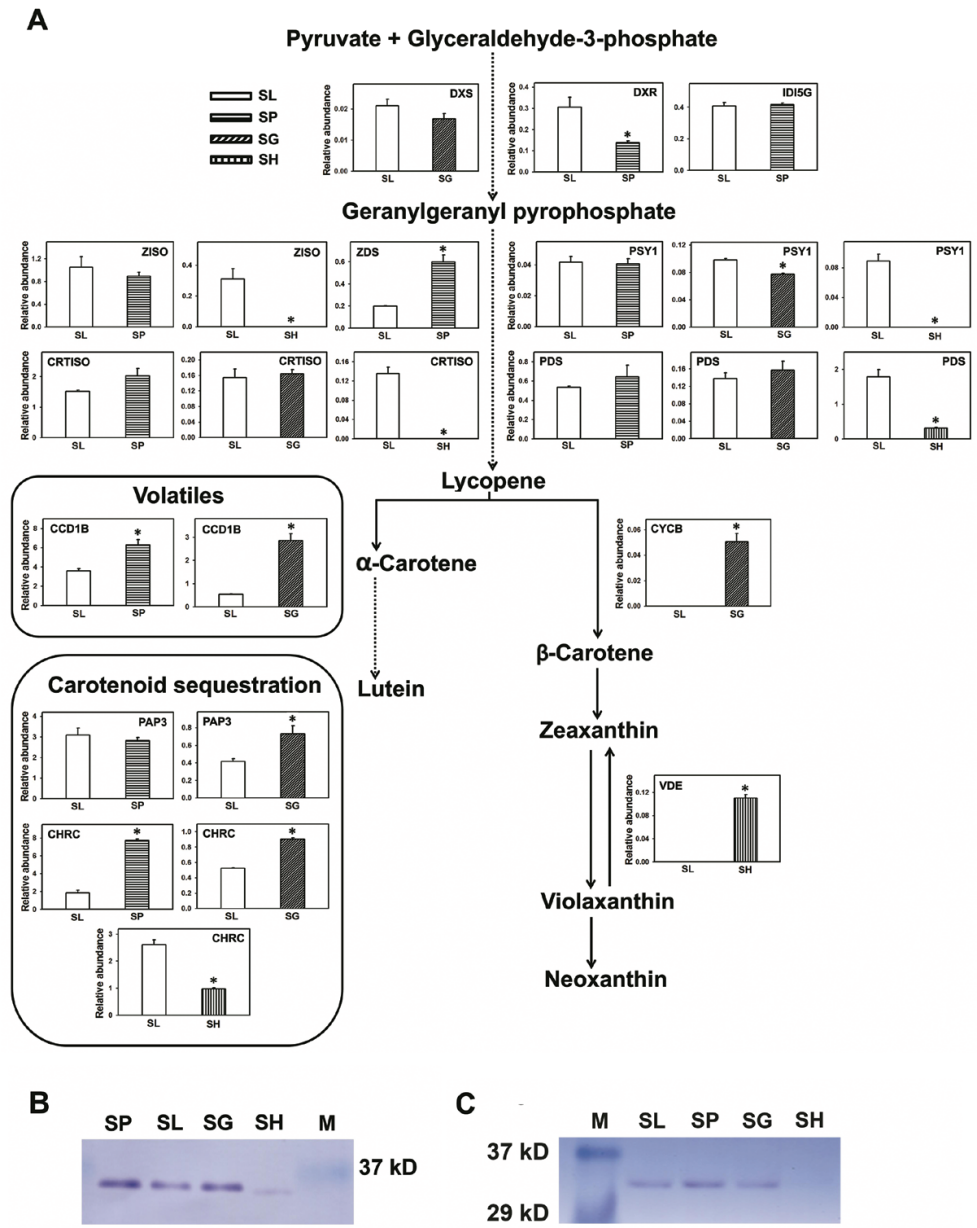
Relative abundances of proteins mediating carotenoid biosynthesis, sequestration and volatile formation in 39 dpa fruits of wild species. Targeted peptide monitoring was employed to quantify the protein abundances relative to internal control proteins. Data are means±SE (*n*=3), *P≤0.05, and presented in Table S7. (A) The relative abundances of proteins involved in carotenoid biosynthesis are represented at respective locations on the pathway. The proteins mediating carotenoid sequestration and cleavage for volatile formation are enclosed in a rectangle. Dotted arrows indicate multiple steps in the pathway. (B, C) For western blots, proteins were extracted and immunoblotted as described in methods. (B) For the detection of CHRC, an equal amount (10 µg) of protein was loaded and probed with the bell pepper PAP antibody. (C) For PAP3 detection, 15 µg of protein was loaded in all the wells and probed with the PGL35 antibody. CCD1B, carotenoid cleavage dioxygenase 1B; CHRC, chromoplast-specific carotenoid-associated protein; CRTISO, carotenoid isomerase; CYCB, chromoplast specific lycopene β-cyclase; DXR, deoxy-xylulose 5-phosphate reductase; DXS, deoxy-xylulose 5-phosphate synthase; IDI5G, isopentenyl diphosphate isomerase 5g; PAP3, plastid lipid-associated protein 3; PDS, phytoene desaturase; PSY1, phytoene synthase 1; VDE, violoxanthin de-epoxidase; ZDS, ζ-carotene desaturase; ZISO, ζ-carotene isomerase.

Consistent with high lycopene level, TPM revealed up-regulation of ZDS, CHRC, and CCD1B in SP (Fig. 7A), whereas deoxy-xylulose 5-phosphate reductase (DXR) showed down-regulation and no differential accumulation of IDI5G, PSY1, phytoene desaturase (PDS), or CRTISO was observed compared with SL. Comparison of protein *versus* transcript levels in SP showed significant correlation only for PDS, ζ-carotene isomerase (ZISO) and PAP3 (see Supplementary Table S7). The absence of protein–transcript correlation for other proteins indicated that their accumulation may be modulated post-transcriptionally. A post-transcriptional regulation of carotenogenesis was also observed by Fraser *et al.* (2007) for PDS, ZDS and CRTISO in a *psy1* overexpressor line of tomato. As SG fruits prominently accumulate β-carotene, chromoplast specific lycopene β-cyclase (CYCB) was detected only in SG. In SG too, only a few proteins showed down-regulation (PSY1) and up-regulation (CCD1B, PAP3, and CHRC). Both positive (deoxy-xylulose 5-phosphate synthase (DXS), PSY1, PAP3, CHRC) and negative (CYCB) correlations were found between proteins *versus* transcripts levels (Supplementary Table S7).

The detection of relatively few proteins in SH–SL is likely related to the modified extraction protocol and low protein abundance. Interestingly proteins contributing to the lycopene pathway (PSY1, ZISO, CRTISO) were detected only in SL except PDS, whereas violoxanthin de-epoxidase (VDE) was detected only in SH. Compared with SL, PDS (5-fold) and CHRC (2.7-fold) were down-regulated in SH. In contrast to SG and SP, reduction in the abundance of CHRC in SH is likely related to the absence of lycopene and low levels of β-carotene. Comparison of proteins *versus* transcripts levels showed no significant correlations between any protein–gene pairs in SH (Supplementary Table S7).

We next ascertained whether classical western blot analysis confirmed the protein levels determined by TPM using CHRC and PAP3, as antibodies against these proteins are available. Consistent with the TPM, CHRC levels were in the order SP>SG>SL>>SH (Fig. 7B) and PAP3 levels were in the order of SP≥SL=SG and below the level of detection in SH (Fig. 7C).

### Heterologous expression of SH PSY1 shows normal enzyme activity while CYCB is inactive

Considering that SH fruit retains photosynthesis, and lacks lycopene and preferential accumulation of β-carotene, a critical question is whether genes for fruit-specific carotenogenesis are functional in these fruits. To address this question, we examined whether the rate-limiting enzymes of this pathway, PSY1 and CYCB, are active using a functional complementation assay. We expressed the full-length *psy1* cDNA and *cycb* cDNA from SL and SH in *E. coli* carrying the plasmids pAC-85b and pAC-LYC, respectively.

**Fig. 8.**
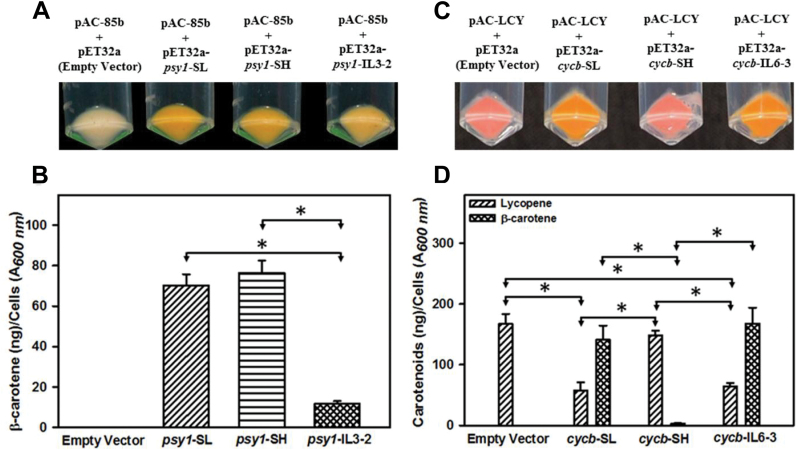
Functional complementation of *psy1* and *cycb* cDNA. (A) *E. coli* cells having pAC-85b plasmid were transfected with empty vector, pET32a-*psy1*-SL, pET32a-*psy1*-SH and pET32a-*psy1*-IL3-2 constructs. β-Carotene formation was observed only in the cells transformed with *psy1* cDNA. (B) β-Carotene levels in the transformed and empty vector cells. Data are means±SE (*n*=3); *P≤0.001 between samples indicated by arrowheads. (C) *E. coli* cells having pAC-LYC plasmid were transfected with empty vector, pET32a-*cycb*-SL, pET32a-*cycb*-SH and pET32a-*cycb*-IL6-3 constructs. β-Carotene formation was observed only in the cells transformed with a functional *cycb* cDNA. (D) Lycopene and β-carotene levels in the transformed and empty vector cells. Data are means±SE (*n*=3); *P≤0.001 between samples pointed by arrowheads. UHPLC profiling of carotenoids and estimation was carried out as per the protocol described in Gupta *et al.* (2015). (This figure is available in color at *JXB* online.)

The *E. coli* cells carrying pAC-85b plasmid lack *psy1* and form β-carotene only upon complementation with a functional *psy1* gene (Cunningham and Gantt, 2007). The transfection of bacteria with pET32a-*psy1*-SL and pET32a-*psy1*-SH constructs led to orange cells (Fig. 8A) due to the accumulation of high β-carotene levels (Fig. 8B and Supplementary Fig. S8A–E). The above complementation indicated that the *psy1* gene of SH encodes a functional phytoene synthase. Considering that in another green-fruited species, *S. pennellii*, the *psy1* gene is purported to be functionally inactive (Bolger *et al.*, 2014), we examined its activity by functional complementation using IL3-2 fruits harboring an *S. pennellii* chromosomal segment of the *psy1* gene. In contrast to SH, *E. coli* cells transfected with pET32a-*psy1*-IL3-2 showed pale orange color with substantially lower β-carotene compared with SL or SH *psy1*-complemented cells. Ostensibly, though both SH and *S. pennellii* are green-fruited, the former encodes a functional PSY1, whereas the PSY1 function is reduced in IL3-2 (Fig. 8B). We, therefore, examined whether IL3-2 fruits express *psy1* like SH. In IL3-2 fruits, *psy1* expression was significantly lower than SH though it was similar to SL (see Supplementary Fig. S8C).

The SH fruits showed *cycb* expression slightly higher than SL, whereas consistent with *S. pennellii* (Bolger *et al.*, 2014), the expression of *cycb* in IL6-3 fruits was much higher than SL or SH (see Supplementary Fig. S8F). Compared with SL, SH *cycb* harbored four and *S. pennellii* harbored 12 non-synonymous single nucleotide polymorphisms (SNPs), of which E244D in SH was predicted to be deleterious by the sorting intolerant from tolerant (SIFT) algorithm (see Supplementary Fig. S8G, H). To ascertain whether CYCB function in SH and *S. pennellii* is also affected, we used the pAC-LYC plasmid harboring genes necessary for β-carotene formation except *cycb*. The *E. coli* cells transfected with this plasmid form β-carotene when complemented with a functional *cycb* gene; otherwise they form only lycopene (Cunningham *et al.*, 1994). The bacteria transfected with pET32a-*cycb*-SL formed orange colonies, whereas pET32a-*cycb*-SH transfected bacteria formed red colonies (Fig. 8C). The carotenoid profiling of the above bacteria revealed both lycopene and β-carotene for SL. In contrast, for SH, bacteria formed lycopene equal to empty vector control and only trace levels of β-carotene (Fig. 8D; Supplementary Fig. S8I, J) suggesting that the SH *cycb* gene is only marginally functional. Contrarily, *E. coli* cells transfected with the *cycb* gene from IL 6-3 formed orange cells, and both lycopene and β-carotene were detected in cells analogous to SL (Fig. 8C, D). From the foregoing, it is apparent that the lack of fruit-specific carotenogenesis in SH fruits may partially stem from the absence of functionally active CYCB.

## Discussion

In the tomato clade, green-fruited species are considered more primitive than orange- and red-fruited relatives (Aflitos *et al.*, 2014). Although they all share the same gene repertories for carotenogenesis, little is known about shifts in carotenogenesis governing transition from green- to orange/red-colored fruits. In this study, we used information from carotenoid analysis, carotenogenic gene expression, and proteome characterization to reveal the blocks that preclude ripening-specific carotenoid formation in SH.

### Green to red fruit transition involved shift towards the β-branch of carotenogenesis

The variation in fruit coloration in the tomato clade signifies that the carotenoid biosynthesis is uniquely regulated in each species. Characteristic accumulation of β-carotene in SG, high lycopene in SP and no preferential accumulation of β-carotene and lycopene in SH support this contention. Apparently fruit coloration in the tomato clade underwent two transitions; first, high β-carotene accumulation in SG, and second, high lycopene accumulation in SP and SL. The transition to the β-carotene pathway is also consistent with the enlisting of a chromoplast-specific lycopene-β-cyclase (CYCB) to produce β-carotene. In SG, preferential up-regulation of the β-carotene branch was associated with attenuation of the α-carotene branch. The β-branch was also favored in red-fruited species, SP and SL, though lycopene is the major carotenoid. This shift to β-carotene is missing in SH where both α- and β-carotene branches contribute almost equally. Moreover, the persistence of neoxanthin, a leaf-specific carotenoid, in SH fruits indicates the operation of a leaf-specific pathway, whereas disappearance of antheraxanthin, similar to SG and SP, indicates a partial operation of a fruit-like pathway. Ostensibly, carotenogenesis in SH fruits appears to be at the crossroads of leaf and fruit-specific carotenogenesis.

### Ethylene emission is not correlated with fruit-specific carotenogenesis in wild species

Carotenogenesis in tomato fruits is preceded by a climacteric rise of ethylene. In contrast, though SH fruits emit a high level of ethylene, they do not exhibit fruit coloration. In introgression lines of SH, QTL 12A regulating high ethylene emission from fruits co-segregates with *aco1* (Dal Cin *et al.*, 2009). Consistent with this, SH fruits show up-regulation of *aco1* and *aco3* genes and also higher levels of an ACO isoform at 29 dpa. Interestingly, even in SP and SG, fruit-specific carotenogenesis does not appear to be correlated with high ethylene emission (Grumet *et al.*, 1981). Considering that all wild relatives emit higher ethylene than SL, the transition of green-fruited species to red-fruited SL appears to be accompanied by attenuation of ethylene biosynthesis.

### Proteome complement related to photosynthesis is retained in SH fruits

The inability to accumulate carotenoids in SH fruits may stem from the absence of chloroplast to chromoplast transformation. Consistent with this, SH fruits retain thylakoid grana, have few plastoglobules, and lack undulating membranes enriched with lycopene crystalloids. Ripe fruits of tomato *gf* mutant, with a lesion in the *stay green* (*sgr*) gene, show similar retention of thylakoids (Cheung *et al.*, 1993; Barry *et al.*, 2008). Similar to SH, *gf* fruits also exhibit impaired chlorophyll degradation and retain photosynthetic proteins (Cheung *et al.*, 1993; Akhtar *et al.*, 1999).

The examination of the proteome complement revealed the persistence of photosynthesis-related proteins in SH fruits. A broader comparison of the different protein functional classes at 39 dpa revealed retention of PS Calvin cycle (8.6%) and light reaction (8.6%) proteins in SH compared with SL (see Supplementary Table S8). In contrast, in SG a minor retention (1.3%) and in SP no retention of these classes was observed. The retention of the above proteins in SH is also consistent with the higher expression of photosynthesis-related genes in *S. pennellii* (Bolger *et al.*, 2014). The higher level of proteins such as chlorophyll *a*/*b* binding protein and oxygen evolving enhancer protein 2 is also consistent with higher PSII activity in SH fruits than in SP/SG. Likewise, Calvin cycle proteins, Rubisco activase 1 and fructose bisphosphate aldolase were also at higher levels in SH. Similar to SH, photosynthesis remains active for a long period in mature fruits of *S. pennellii* (Bolger *et al.*, 2014).

The retention of thylakoids in SH chloroplasts is in conformity with the persistence of ATP-dependent filamentation temperature sensitive metalloprotease class (FtsH) proteins in SH fruits. During chromoplast formation, the decrease in the levels of FtsH isoforms is reportedly associated with the disassembly of thylakoids (Barsan *et al.*, 2012). Consistent with this in SP and SG fruits, the levels of FtsH2 and FtsH5 decreased in abundance from 26 to 39 dpa. In contrast, in SH fruits, a differential decrease in FtSH was observed (see Supplementary Table S6). While the abundance of FtsH2 in SH decreased similar to SP/SG, the level of FtsH5 remained stable. Since thylakoid loss takes place only when FtsH2 and FtsH5 levels drop below a threshold in Arabidopsis and tobacco (Kato *et al.*, 2012), it is likely that in SH fruits, high levels of FtsH5 protein at 39 dpa subdues this response.

In concert with thylakoid retention, the SH fruits also show impaired chlorophyll destabilization. In tomato, fruit ripening correlates with the up-regulation of genes mediating the PAO/phyllobillin pathway promoting chlorophyll catabolism (Guyer *et al.*, 2014). In SH fruits, thylakoid disassembly marked by the appearance of chlorophyll catabolism proteins and disappearance of pheophorbide *a* seems to be constrained. The SH fruits accumulate 10-fold higher pheophorbide *a* than SP/SG. The PAO and RCCR proteins catabolizing pheophorbide *a* were not detected in SH, though these were present in SG and SP. Likewise, the coupled assay of PAO–RCCR showed much lower activity in SH than SL. From the foregoing, it appears that unlike the orange/red-fruited species, SH fruits lack the proteome-shifts associated with chromoplast formation.

### Expression of genes influencing carotenogenesis is not compromised in SH

Among the genes regulating the MEP pathway, expression of *dxs* seems to be critical for regulating GGPP, a primary precursor for the carotenoid and isoprenoid pathway (Lois *et al.*, 2000; Enfissi *et al.*, 2005; Ruiz-Sola and Rodríguez-Concepción, 2012). Considering that all wild relatives exhibited higher expression, *dxs* does not appear to be a limiting factor for carotenoid formation in SH.

In tomato, fruit-specific carotenogenesis is associated with the up-regulation of *psy1* and *cycb* genes. Therefore, green-fruitedness of SH could be due to non-functional *psy1* and/or *cycb* genes or their subdued expression. It has been suggested that absence of *psy1* up-regulation likely imparts green and yellow fruit colors in the tomato clade (Meléndez-Martínez *et al.*, 2010; Bolger *et al.*, 2014). Though the *psy1* expression in SH was relatively higher than in SL, yet its fruits do not accumulate phytoene and phytofluene similar to SP and SG fruits at 39 dpa.

Likewise, high *cycb* expression in SH fruits does not increase β-carotene levels. A similar mismatch between *cycb* expression and carotenoid accumulation is also observed in *S. pennellii* (Bolger *et al.*, 2014). Considering that *psy1* and *cycb* mediate two key regulatory steps in SL coloration, it appears that carotenogenesis in SH is probably not compromised by lower gene expression at least at these two steps.

### SH encodes a functional PSY1 and a non-functional CYCB

In green-fruited wild relatives such as *S. pennellii*, the lack of lycopene accumulation is attributed to the diminished expression of the *psy1* transcript (Meléndez-Martínez *et al.*, 2010; Bolger *et al.*, 2014). It has been assumed that the presence of an SNP in *S. pennellii* is responsible for the block in PSY1 function (Bolger *et al.*, 2014). Considering that SH and *S. pennellii* share the same non-synonymous SNP leading to A408V substitution in PSY1 compared with SL (see Supplementary Fig. S8D, E), it is logical to expect that PSY1 of SH may be non-functional as in *S. pennellii*. Contrarily, the functional complementation of SH *psy1* cDNA showed normal PSY1 activity, restoring β-carotene formation in *E. coli* harboring pAC-85b. Though SH PSY1 is functional, the SH fruits do not accumulate PSY1 protein to a detectable level. Since SH fruits show considerable expression of the *psy1* transcript, it appears that the below-detection levels of PSY1 protein may result from either reduced protein translation or enhanced degradation. The absence of phytoene and phytofluene accumulation is also in conformity with the low abundance of PSY1 in SH fruits. Interestingly, *psy1* cDNA cloned from the IL3-2 line displayed reduced activity, indicating that in *S. pennellii*, PSY1 activity may be affected by SNP(s) other than the one contributing to A408V substitution. The existence of a functional phytoene synthase indicates that in green-fruited SH, the block in carotenogenesis is located at loci other than *psy1*.

In addition to *psy1*, the expression of the *cycb* gene is also important for fruit coloration in the tomato clade. Consistent with higher accumulation of β-carotene in SG, CYCB protein was detectable only in SG and not in SL, SP, or SH. Considering that SH encodes a functional PSY1, it may similarly encode a functional CYCB. However, functional complementation of SH *cycb* cDNA indicated that SH CYCB is barely active, while SL encodes an active CYCB. The lack of CYCB activity in SH could be attributed to the presence of SNPs (see Supplementary Fig. S8G, H). Among the SNPs present in SH relative to SL, the E244D substitution may have rendered it non-functional, as SIFT analysis predicted this change to be deleterious to the protein function (SIFT score 0.012; Vaser *et al.*, 2016). Contrary to the non-functional CYCB in SH, *cycb* cDNA from IL6-3 harboring an *S. pennellii* chromosomal segment (Liu *et al.*, 2003; Bolger *et al.*, 2014), restored the formation of β-carotene in a complementation assay. Compared with SL, *S. pennellii cycb* has 12 amino acid substitutions, but only one of these (T3A, SIFT score 0.018) is predicted to be deleterious by SIFT. However, since this SNP is located in the transit peptide, it may not have any effect on CYCB function. Notwithstanding the presence of a deleterious SNP in transit peptide, the acquisition of normal fruit coloration in IL 6-3 is consistent with *S. pennellii* encoding a CYCB that is functional at least in the SL background.

### Key proteins needed for carotenogenesis/storage are not detected in SH fruits

The regulation of carotenogenesis in tomato fruits, so far, is mainly inferred from transgenic approaches and using mutants compromised in fruit-specific carotenogenesis. Currently only limited information is available about the *in vivo* level/activity of proteins contributing to carotenogenesis due to their very low abundance (Barsan *et al.*, 2012; Kilambi *et al.*, 2013; Wang *et al.*, 2013). The use of TPM enabled us to detect and quantify several carotenogenic proteins in tomato and its wild relatives.

In tomato fruit-specific carotenogenesis, the first precursor phytoene is exclusively synthesized by a fruit-specific PSY1 (Fray and Grierson, 1993), with no proven role for leaf-specific PSY2 (Fraser *et al.*, 1999). Consistent with enhanced carotenoid levels, PSY1 was detected in SP and SG but not in SH fruits. The absence of PSY1 in SH fruits may be due to its low abundance and perhaps contributes to the absence of lycopene/β-carotene accumulation. As proteins contributing to a metabolic pathway tend to be coordinately regulated, it is possible that their abundance may be similarly determined. Accordingly, the abundance of proteins contributing to lycopene formation was higher in SL and SP fruits. Similarly, increased abundance of CYCB may relate to high β-carotene level in SG fruits. The levels of carotenoids in fruit are not static as a portion is channelized for emission of volatiles. Consistent with the fact that the carotenoid-derived aroma volatiles are largely emitted from ripened fruits (Rambla *et al.*, 2014), CCD1B was detected in SG and SP, but not in SH fruits.

Accumulation of carotenoids also depends on sequestration facilitated by proteins like CHRC and PAP3 (Kilambi *et al.*, 2013; Nogueira *et al.*, 2013). Consistent with this, *high pigment* mutant fruits that are enriched in carotenoids also show an increase in CHRC levels (Kilambi *et al.*, 2013). In conformity with high carotenoid levels, the abundance of CHRC was high in SP fruits. On the contrary, the CHRC levels were diminished in SH fruit, as it accumulates little carotenoids. In addition to CHRC, PAP3 level as detected by immunoblotting was below the detection limits in SH. Lower levels of these proteins entail that the carotenoid sequestration may be compromised in SH fruits.

### Fruit-specific carotenogenesis in SH is blocked at multiple levels

In green-fruited *S. pennellii* the absence of carotenoid accumulation is attributed to a subdued expression of *psy1* (Bolger *et al.*, 2014). Likewise, tomato *psy1* mutants also show the absence of lycopene accumulation (Kachanovsky *et al.*, 2012). It is intriguing that despite having a functional PSY1, SH fruits do not accumulate lycopene. In SL, the mutants defective in the *cycb* gene predominantly accumulate lycopene in the ripening fruits and lack β-carotene (Ronen *et al.*, 2000). Considering this, it is logical that even though SH encodes a non-functional CYCB, its fruits should have accumulated lycopene. One of the reasons for the absence of lycopene could be the low abundance of PSY1 in SH as its fruits do not accumulate phytoene and phytofluene. However, the lack of lycopene accumulation may also stem from the absence of a sink in SH fruits to store the lycopene, which is also corroborated by the dearth of carotenoid sequestration proteins. Therefore, it is reasonable to assume that low abundance of PSY1 along with the absence of carotenoid sequestration and chromoplast formation in SH precludes lycopene accumulation.

In summary, this study provides evidence that fruit-specific carotenogenesis in green-fruited SH is blocked at multiple levels. The absence of chloroplast to chromoplast transformation, retention of photosynthesis, incomplete degradation of chlorophyll, low abundance of PSY1 protein (though the protein is functional), lack of functional CYCB, and the dearth of carotenoid sequestration proteins collectively contribute towards the green-fruitedness of SH. The current study also entails that overcoming of multitudinous structural, functional, and regulatory blocks governed the transition from green- to orange/red-fruited coloration in the tomato clade. The systems-based approaches in future may help to decipher the molecular basis of these multiple blocks and interactions among them.

## Supplementary data

Supplementary data are available at *JXB* online.

Fig. S1. Experimental design used in the study.

Fig. S2. Fruit phenotypes of tomato and wild relatives during development.

Fig. S3. GeLC-MS scheme used for proteome analysis.

Fig. S4. Workflow for setting up targeted peptide monitoring for quantification of proteins.

Fig. S5. Relative expression levels of regulatory genes in fruits of tomato and wild species.

Fig. S6. Functional classification of differentially expressed proteins in the wild species.

Fig. S7. Absorbance, fluorescence spectra and peak areas obtained after PAO/RCCR assay of 39 dpa fruit extracts of SL and SH.

Fig. S8. Functional complementation of *psy1* and *cycb* in *E. coli*.

Table S1. Primers used in this study for quantitative real-time PCR and functional expression of *psy1* and *cycb* in *E. coli*.

Table S2. Peptides used for targeted proteomic monitoring of carotenogenic proteins in 39 dpa fruits of tomato and wild species.

Table S3. Internal control proteins and peptides used for targeted proteome monitoring.

Table S4. Differentially expressed proteins identified in the fruits of wild species at different developmental stages using nanoLC-MS-MS.

Table S5. Proteins identified only in wild species but not in tomato.

Table S6. Abundance patterns of proteins involved in chromoplast formation in the fruits of wild species and tomato.

Table S7. Carotenoid, transcript, and protein abundances in fruits of wild species and tomato.

Table S8. Comparison of the percentage of differentially expressed proteins based on functional classification in the 39 dpa fruits of tomato and wild species.

## Author contributions

The whole study was conceived and designed by YS. HVK performed transcript profiling, proteome analyses, ethylene evolution, transition electron microscopy, Hill reaction, and PAO assay; KM performed TPM; KM and HVK performed all other mass spectrometric identifications; functional expression of *psy1* and *cycb* was done by AR, carotenoid profiling was done by CC and JB, western blotting and SNP analysis was done by CC. Plants were grown, tagged and maintained by HVK and JB. All the data were analysed by HVK, KM, AR, CC, and YS. YS and RS wrote the manuscript with inputs from HVK, KM, CC, and AR. All authors read and approved the manuscript.

## Supplementary Material

supplementary_table_S1

supplementary_table_S2

supplementary_table_S3

supplementary_table_S4

supplementary_table_S5

supplementary_table_S6

supplementary_table_S7

supplementary_table_S8

supplementary_figures_S1_S8

## Abbreviations:

ACO: 1-aminocyclopropane-1-carboxylic acid oxidase
CCD1B: carotenoid cleavage dioxygenase 1B
CHRC: chromoplast-specific carotenoid-associated protein
CRTISO: carotenoid isomerase
CYCB: chromoplast specific lycopene β-cyclase
dpa: days post-anthesis
DXS: deoxy-xylulose 5-phosphate synthase
GGPP: geranylgeranyl diphosphate
MEP: methylerythritol-4-phosphate
PAO: pheophorbide *a* oxygenease
PAP: plastid lipid-associated protein
PDS: phytoene desaturase
PSY: phytoene synthase
RCCR: red chlorophyll catabolite reductase
SG: *S. galapagense*
SH: *S. habrochaites*
SL: *S. lycopersicum* cv. Ailsa Craig
SP: *S. pimpinellifolium*
TPM: targeted peptide monitoring
ZDS: ζ-carotene desaturase;
ZISO: ζ-carotene isomerase.
