# Supplementary material for: Green-fruited Solanum habrochaites lacks fruit-specific carotenogenesis due to metabolic and structural blocks
Source: J Exp Bot. 2017 Oct 9;68(17):4803–19. doi: 10.1093/jxb/erx288 (PMC5853803; doi:10.1093/jxb/erx288)
Supplement: supplementary_table_S1 [file erx288_suppl_supplementary_table_s1.pdf]

Table S1. A. Primers used for quantitative real-time PCR analysis in this study.

Gene	Forward primer (5′ to 3′)	Reverse primer (5′ to 3′)	Product size (bp)
dxs (Solyc01g067890.2.1)	AAATGGGATCGGTGTAGAGC	TGCTGAGCCATATCCCAATA	115
dxr (Solyc03g114340.2.1)	AGCAGGGTGTGATTGAGGTT	GTCTTTTCCTGCTTCGATGG	113
hdr (Solyc01g109300.2.1)	TACGGGATGCCTAAGGCTAA	TTCACCACATGCTTTTCTCG	112
idi5g (Solyc05g055760.2.1)	CCAGACGAAGTTGCTGACAT	TCCCACCATTTGAACAAGAA	143
idif (Solyc08g075390.2.1)	GCCTTTACTCTCCAGGAATCAG	ATTCCCCATTCACATCATAAGG	118
idi4g (Solyc04g056390.2.1)	GAGAGGAATTGATGGGAACAAG	GAAGTTATAGGGGAGGCGATG	93
ggpps2 (Solyc04g079960.1.1)	ATCAATGGAGCAGCTTTGTG	GCGGTTGATAAAACGACGTA	128
ggpps2g (Solyc02g085700.1.1)	AATTTGCAAGATGTATTGGTTTG	AAGTCCTTCCCAGCTGTTTTC	100
ggpps9g (Solyc09g008920.2.1)	AAAGAAAACTTGGCCGTTATTG	TCTAGGCAATGAAACCAAACTG	100
ggr (Solyc03g115980.1.1)	TTACTGGCCAACATACAAGGTG	ACATACTCATCAGCGCACATCT	102
psy1 (Solyc03g031860.2.1)	TGAATTAGCACAGGCAGGTC	TCAATTCTGTCACGCCTTTC	140
psy2 (Solyc02g081330.2.1)	AATTCCGAGGTCTCATACGG	CCTTTCCACATCGAATTCCT	110
pds (Solyc03g123760.2.1)	TATCATCAACGTTCCGTGCT	TATCGGTTTGTGACCAGCAT	122
ziso (Solyc12g098710.1.1)	AGAGCGTGCTTTTCGTGTATTG	ATTGCCATAACTGCACTCCATC	107
zds (Solyc01g097810.2.1)	TCCAAAAGGGCTATTTCCAC	TTGATCCAAGAGCTCCACAG	115
crtiso (Solyc10g081650.1.1)	GAGATCGCCAAATCCTTAGC	CAGAAAGCTTCACTCCCACA	118
lcyb1 (Solyc04g040190.1.1)	CGATGCAACTGGCTTCTCTA	AATGAGAATCTCGCCAATCC	149
lcyb2 (Solyc10g079480.1.1)	ATTTGTGGCCCATAGAAAGG	TGACAAGAAACCATGCCAAT	146
cycb (Solyc06g074240.1.1)	TCTTCTCAAGCCTTTTCCATC	TGGTGGGACTTAGAAAAGAAGG	92
lcye (Solyc12g008980.1.1)	TTAGTCGCCATTTTCTGCAC	TCACCCTCGCACTCTACAAG	130

crtrb1 (Solyc06g036260.2.1)	CAAACGCTGTTCCAGCAATA	TCTTGTGAACCAAACCATCG	141
crtrb2 (Solyc03g007960.2.1)	CGCCATAACAAATGCTGTTC	ATGAACCAGTCCATCGTGAA	148
zep (Solyc02g090890.2.1)	GGTCGTGTTACATTGCTTGG	TGCATGCTTTTTCAAGTTCC	118
vde (Solyc04g050930.2.1)	GTGCAGCTAATGTTGCCTGT	GGGAGACTGCACACTCATTG	126
nxs (Solyc12g041880.1.1)	GATGAGCTTGTGGTGATTGC	ACAACCTTCCGTCCATGAAG	113
cyp97a29 (Solyc04g051190.2.1)	GTGCCATTGTACCAGCATTG	TGCAGCAACATCAAGCTTTT	101
cyp97c11 (Solyc10g083790.1.1)	TGCTGAGAGAATGGTGGAGA	GTGCAAGGCCAATAACATCA	107
pap3 (Solyc08g076480.2.1)	AAGAAGTGTGCTGAAATAAAATGTG	AAGCTGAAATTACAGATGATTGACC	104
chrc (Solyc02g081170.2.1)	ACCAATAGGGGTTTGAGTGC	GCCGTTGAGAAGAGTCAAGG	120
nor (Solyc10g006880.2.1)	GGCAATATTCGGAGAGCAAG	TTCCGGTAGCCTTCCAATAA	113
rin (Solyc05g012020.2.1)	ATGGCATTGTGGTGAGCAAAG	GTTGATGGTGCTGCATTTTCG	147
cnr (Solyc02g077920.2.1)	AAATTTGGGCTGAAGAAGCA	AATCCGGGAATTGACAGAAG	115
tagl1 (Solyc07g055920.2.1)	CAGCCAAATTACGAAGATGC	CAAGCTGGAGAGGAGTTTGG	109
Or (Solyc03g093830.2.1)	GGAGTTGCAGGAAATTCGAG	ATCCCAAGCTCAGCACTCTT	104
erf6 (Solyc01g065980.2.1)	TCCCTACTACCCCATGGAAA	GATCAGATGGCTCCAATTCC	101
ful1 (Solyc06g069430.2.1)	TTGGGGAAGCATACCAGAGT	ATGACGAAGCATCCATTGTG	119
ful2 (Solyc03g114830.2.1)	AGAAAGAGATGGCCCAACAG	TTGGGTATGCTTCCCCTATG	117
aprr2 (Solyc08g077230.2.1)	CTAATCATGTTGCCCCAGGT	GTCTCTGCAGGTTTCCATCC	118
glk2 (Solyc10g008160.2.1)	GGCACTCCTTGTTTTTCTGC	GCAAATCAGAGGCAACTGTG	118
hb1 (Solyc02g086930.2.1)	TTCTATTTAATCTGAAAACGGGAAA	ACAGTAATCTAGCAGGAGTTTCCAA	101
ap2a (Solyc03g044300.2.1)	AATGGATTAGTGGGGAACAAAA	CTCCGGTAAAAGGTAACACCAC	113
acs2 (Solyc01g095080.2.1)	AAGCTTAACGTCTCGCCTGGAT	AGCGCAATATCAACCGTTCCAT	101
acs4 (Solyc05g050010.2.1)	TATTGAAAGCGCGAAAAGGT	CGCAAATCCATCCAACAATA	117
aco1 (Solyc07g049530.2.1)	AAGAGGCAGAGGAAAGTACACA	GGATCACTTTCCATTGCCTTCA	130

aco3 (Solyc07g049550.2.1)	GTTCTTGGAAGTTATACAAACGTAGC	ACACAACAATCACACACACATACAC	80
pao (Solyc11g066440.1.1)	CATCTCAAGGACCTGAAGCTAA	TCATCAGGCCAAACAAAGAGTA	102
rccr (Solyc03g044470.2.1)	GGCTGTTCCACTCTCCTCTG	GAGCAACAGAAGCGTTTTCC	105
β-actin (FJ532351.1)	TGTCCCTATTTACGAGGGTTATGC	CAGTTAAATCACGACCAGCAAGAT	108
ubiquitin3 (X58253.1)	GCCGACTACAACATCCAGAAGG	TGCAACACAGCGAGCTTAACC	110

dxs, deoxy-xylulose 5-phosphate synthase; *dxr*, deoxy-xylulose 5-phosphate reductase; *hdr*, 4-hydroxy-3-methylbut-2-enyl diphosphate reductase; *idi*, isopentenyl diphosphate synthase (isoforms *f* and *5g*); *ggpps*, geranyl geranyl diphosphate synthase (isoforms *2*, *2g* and *9g*); *ggr*, geranyl geranyl reductase; *psy1*, phytoene synthase 1; *psy2*, phytoene synthase 2; *pds*, phytoene desaturase; *ziso*, ζ-carotene isomerase; *crtiso*, carotenoid isomerase; *zds*, ζ-carotene desaturase; *lcyb1*, lycopene β-cyclase1; *lcyb2*, lycopene β-cyclase2; *cycb*, chromoplast specific lycopene β-cyclase; *lcye*, lycopene ε-cyclase; *crtrb1*, β-carotene hydroxylase 1, *crtrb2*, β-carotene hydroxylase 2; *zep*, zeaxanthin epoxidase; *vde*, violoxanthin deepoxidase; *nxs*, neoxanthin synthase; *cyp97a29*, cytochrome P450 carotenoid β-hydroxylase A29; *cyp97c11*, cytochrome P450 carotenoid ε-hydroxylase C11; *pap3*, plastid lipid associated protein 3; *chrc*, chromoplast specific carotenoid associated protein; *nor*, Nonripening; *rin*, ripening inhibitor; *cnr*, Colorless nonripening; *tagl1*, tomato agamous-like 1; *Or*, DnaJ Cys-rich zinc finger domain-containing protein; *erf6*, ethylene response factor 6; *ful1*, fruitful 1; *ful2*, fruitful 2; *aprr2*, Arabidopsis pseudo response regulator 2-like; *glk2*, golden 2-like; *hb1*, HD-Zip homeobox protein1; *ap2a*, apetala 2a; *acs*, 1-aminocyclopropane-1-carboxylate synthase (isoforms *2* and *4*); *aco*, 1-aminocyclopropane-1-carboxylic acid oxidase (isoforms *1* and *3*); *pao*, pheophorbide *a* oxygenase; *rccr*, red chlorophyll catabolite reductase; *β-actin*; *ubiquitin3*.

Table S1. B. Primers used for functional expression of *psy1* and *cycb* in *E. coli* in this study.

Plasmid	Forward primer (5′ to 3′)	Reverse primer (5′ to 3′)
pAC-85b plasmid for *psy1* expression	GCGCTAGCATGACATCGGAACAGATG for SL, SH and IL3-2<br><br>This includes a *Nhe*I site	GCGGATCCTTATCTTTGAAGAGAGGCAG for SL<br><br>GCGGATCCTTATCTTTGAAGAGAGACAG for SH and IL 3-2<br><br>This includes a *BamH*I site
pAC-LYC plasmid for *cycb* expression	CGGATCCATGGAAACTCTTCTCAAGCC for SL, SH and IL3-2<br><br>This includes a *BamH*I site	CGAGCTCTCAAAGGCTCTCTATTGCTAG for SL, SH and IL3-2<br><br>This includes a *Sac*I site
