# Supplementary material for: Green-fruited Solanum habrochaites lacks fruit-specific carotenogenesis due to metabolic and structural blocks
Source: J Exp Bot. 2017 Oct 9;68(17):4803–19. doi: 10.1093/jxb/erx288 (PMC5853803; doi:10.1093/jxb/erx288)
Supplement: supplementary_figures_S1_S8 [file erx288_suppl_supplementary_figures_s1_s8.pdf]

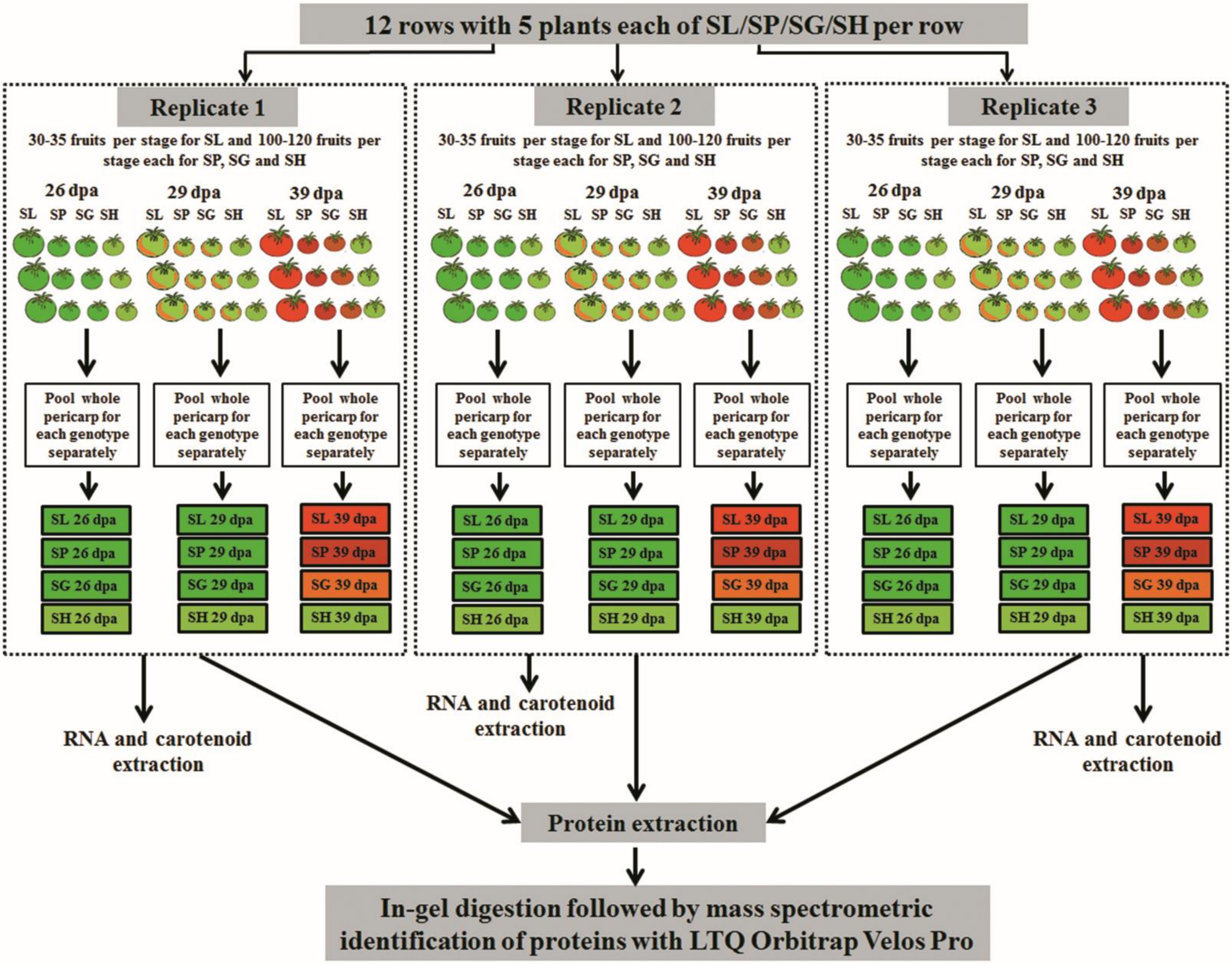

Figure S1. Experimental design describing growth of plant material, collection of fruit tissue at different stages of ripening in tomato and wild relatives for carotenoid, RNA and protein extraction.

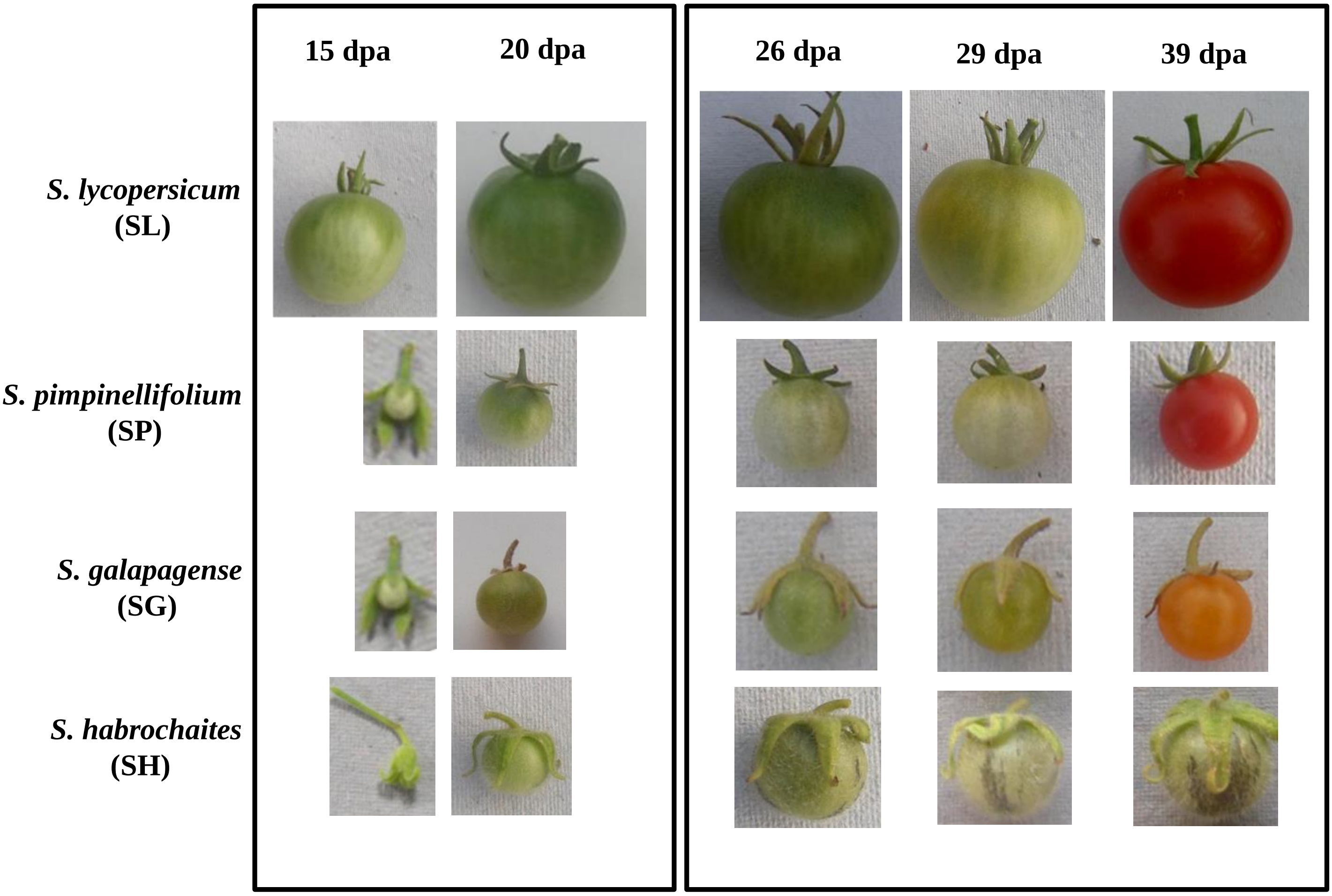

Figure S2. Fruit phenotypes of tomato and wild relatives at different days post-anthesis (dpa) during development. The photographs are not to the scale.

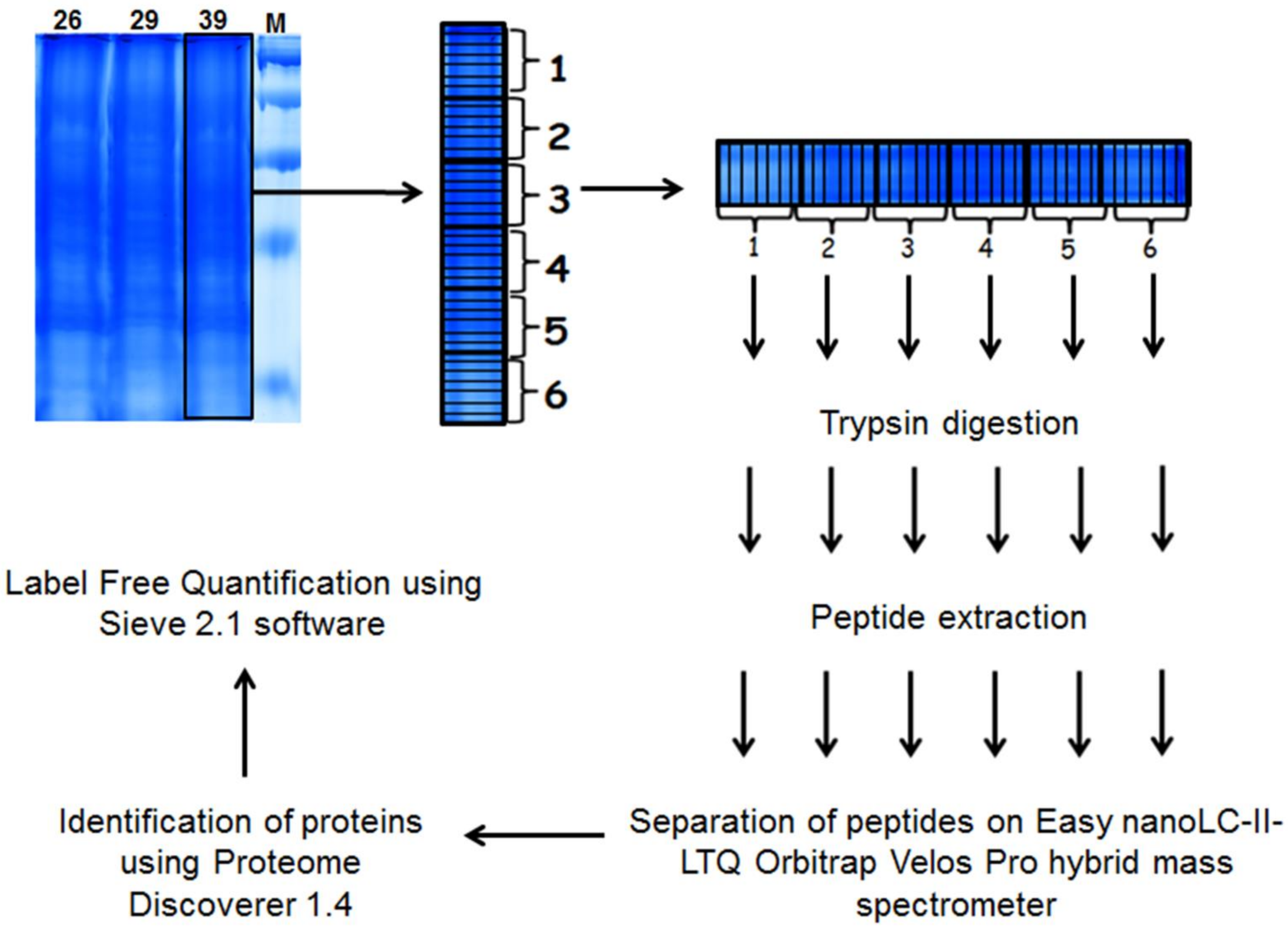

Figure S3. GELC-MS scheme used for proteome analysis in the wild species and tomato.

Workflow for Targeted Peptide Monitoring -

Figure S4

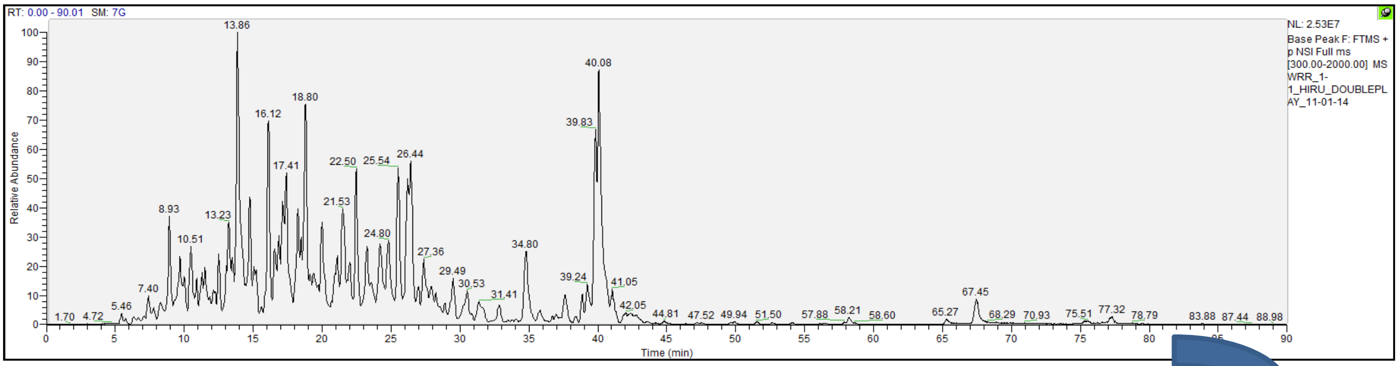

Proteome Discoverer output for Phytoene Desaturase (PDS) protein from SL sample

Proteins | Peptides | Search Input | Result Filters | Peptide Confidence | Search Summary

		Accession	Description	Score	Coverage	# Proteins	# Unique Peptides	# Peptides	# PSMs
1		Solyc03g123760...	functional_description:Phytoene desaturase	7.56	7.55 %	1	2	2	3

	A2	Sequence	# PSMs	# Proteins	# Protein Groups	Protein Group Accessions	Modifications	ΔCn	pRS Probability	pRS Site
1		ASPRPTKPLEIVIAGAGLGGL...	1	1	1	Solyc03g123760.2.1		0.0000		
2		FDFSEALPAPLNGILAILK	2	1	1	Solyc03g123760.2.1		0.0000		

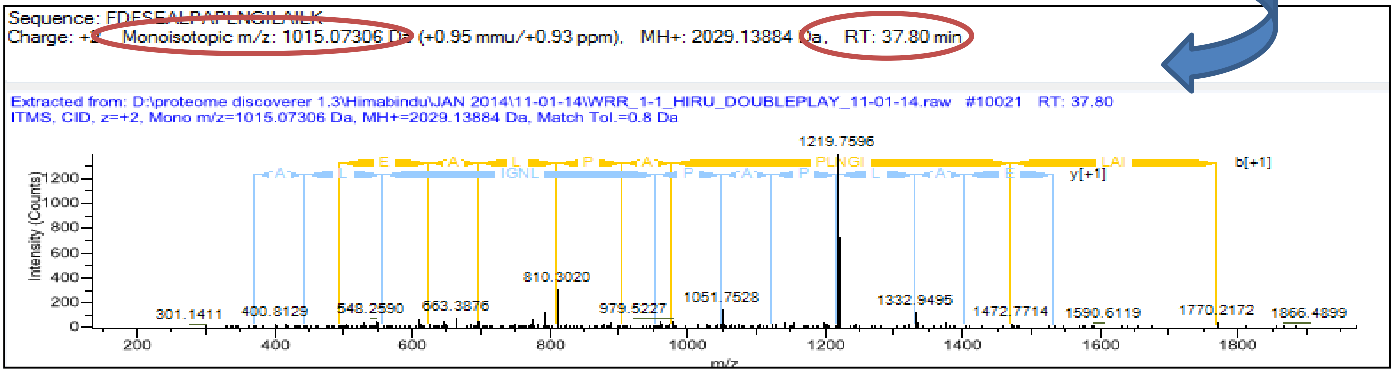

Pictorial representation of a typical TPM experiment -

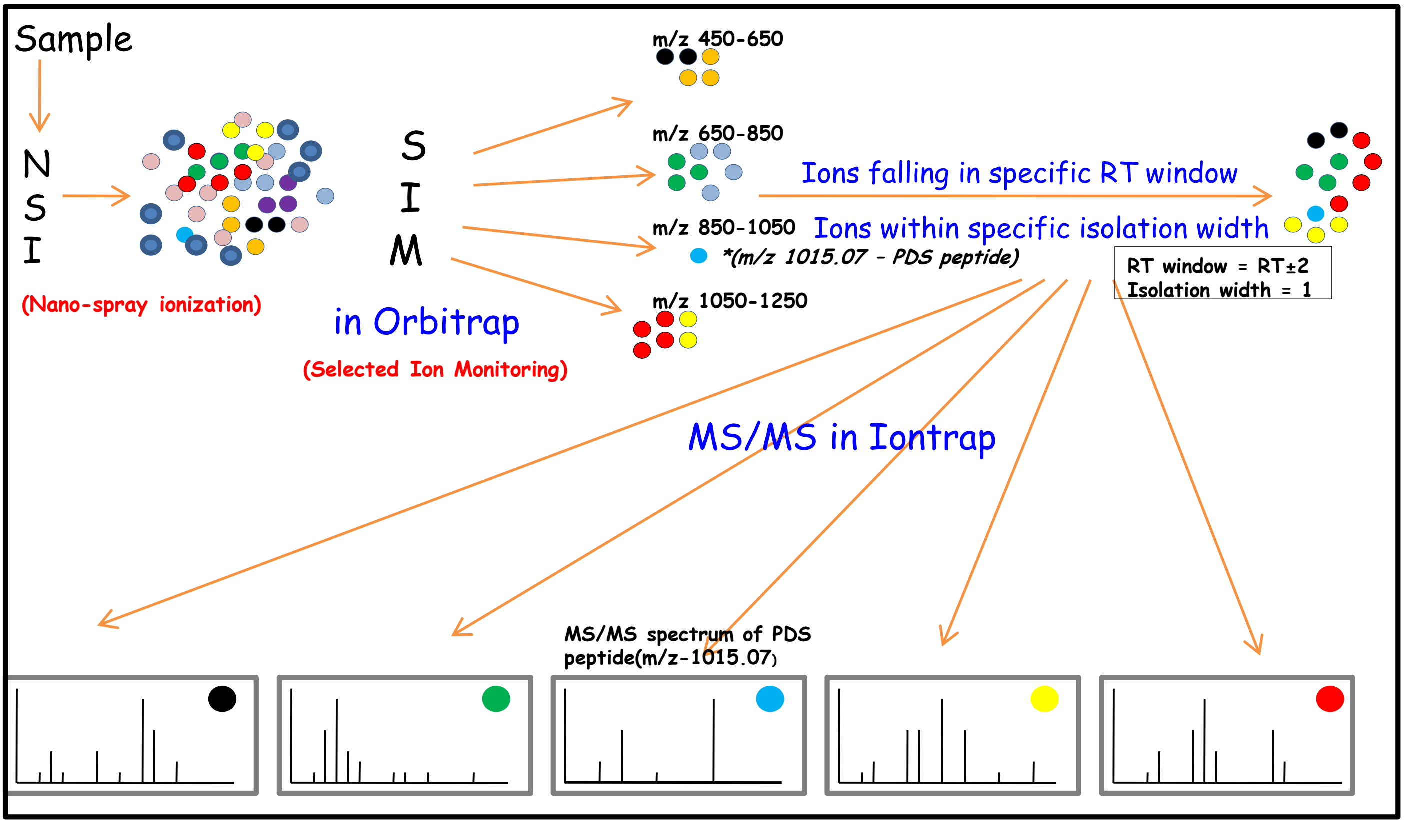

TPM raw file processing -

Figure S4

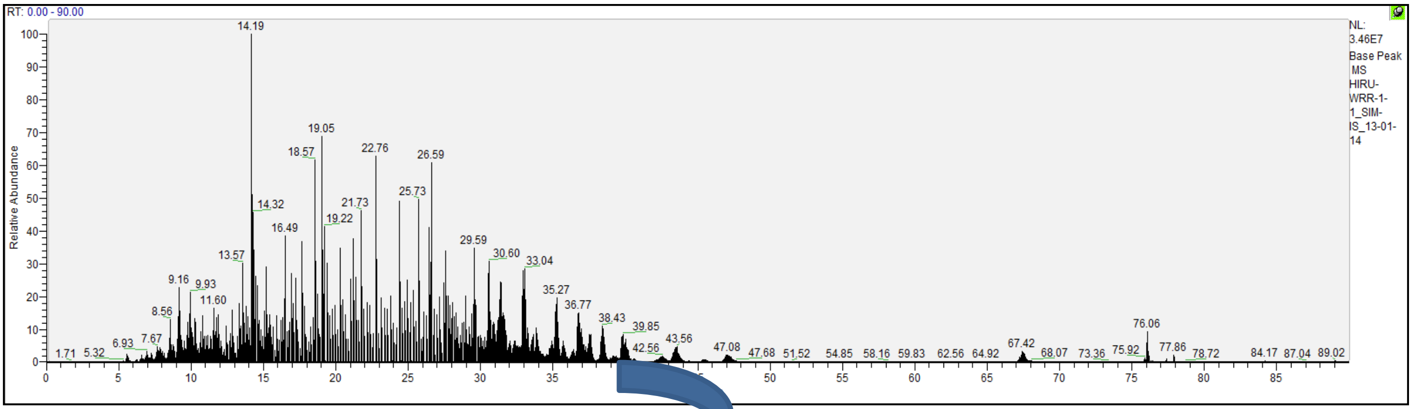

Total Ion Chromatogram of SL sample

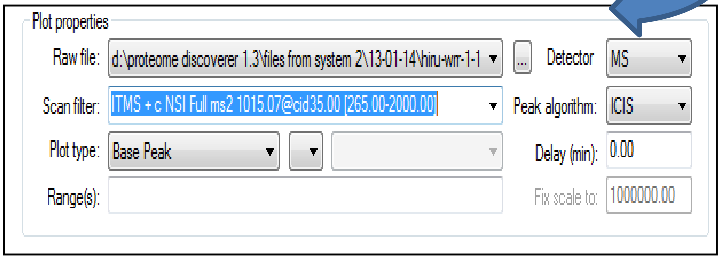

Tab displaying the extraction of m/z 1015.07

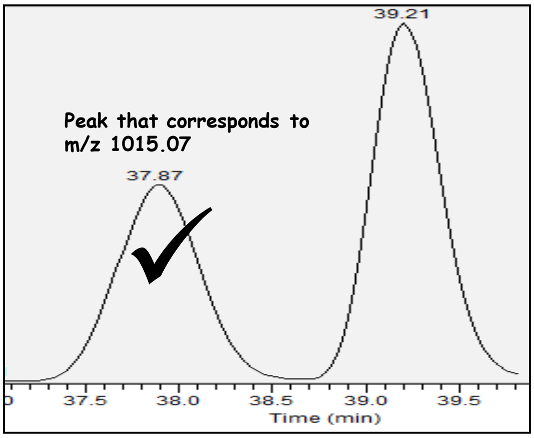

XIC of peak pertaining to m/z 1015.07

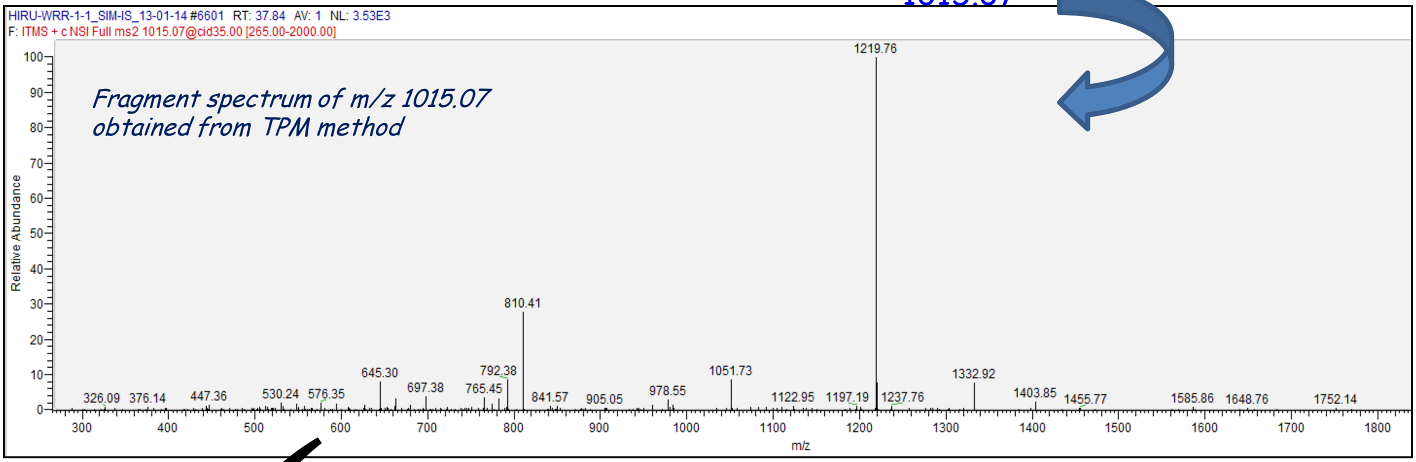

Confirming MS/MS with previous profiling data F D F S E A L P A P L N G I L A I L K

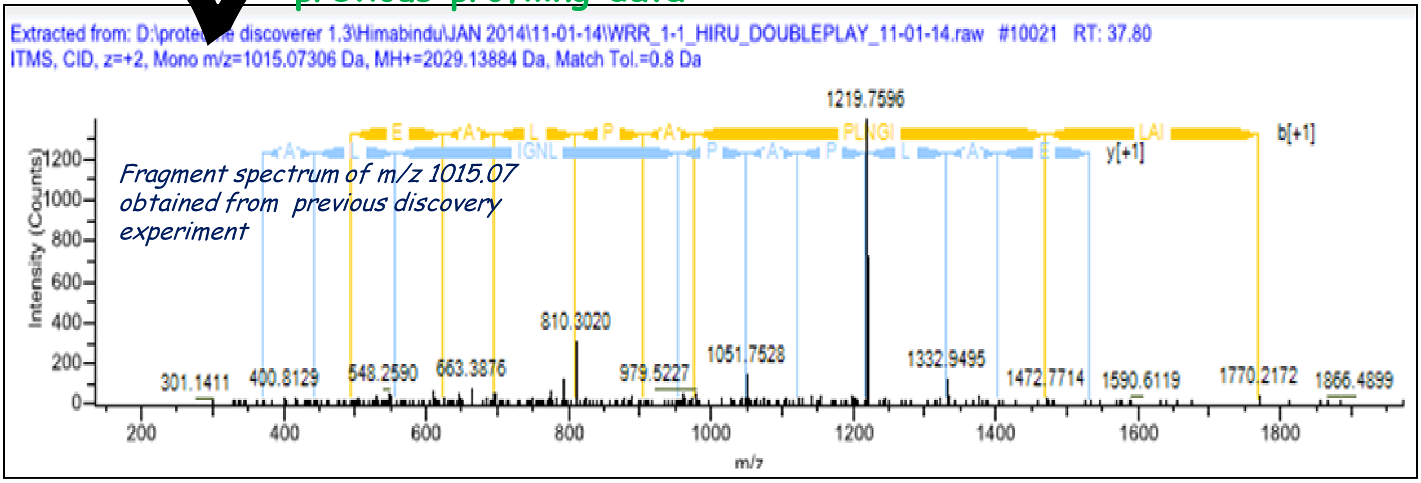

(Similar procedure was carried out for internal reference control proteins)

Calculation of ratio of peak areas –

- Abundance of each pathway protein was represented as a ratio of mean of area under the curve (AUC) of three replicates of peptide of interest and mean of AUC of three replicates of internal reference peptides.

Mean of AUC of peptide = (AUC of 1st replicate+ AUC of 2nd replicate+ AUC of 3rd replicate)/3

Mean of AUC internal reference peptide = (AUC of 1st replicate+ AUC of 2nd replicate+ AUC of 3rd replicate)/3

$$\text{Abundance} = \frac{\text{Mean of AUC of peptide of interest}}{\text{Mean of AUC of internal reference control peptide}}$$

Figure S4. Workflow for setting up targeted peptide monitoring for targeted quantification of proteins. A list of proteotypic peptides for the carotenogenic proteins with precursor m/z values and the expected retention times that were present consistently in all three biological replicates in SL, SP, SG and SH was generated manually. As the use of synthetic labelled peptides was not practically possible for the current study due to the high costs involved, data were normalised with inherent protein controls and validated with retention time standards (Carr *et al.* 2014). For this, Pierce retention time calibration mixture (Thermo Scientific, Product No. 88321, 15 heavy isotope labelled peptides in the mass range of 843.4582-1600.8084) at 50 fmol/sample was used to optimise LC-MS runs. We chose few housekeeping proteins whose abundance remained constant in SL and the wild relatives to serve as inherent controls. Because these internal control proteins undergo similar treatment to the carotenogenic proteins during sample preparation, chromatography, and ionisation, they compensate for any ion suppression effects (Table S3, Sherrod *et al.* 2012).

For TPM, column specifications were same as described in Kilambi *et al.* (2016). Data-independent settings were used for TPM with first four selected ion monitoring (SIM) scans in Orbitrap at a resolution of 30,000 with m/z scan range for each event - 450-650, 650-850, 850-1050 and 1050-1250; the last scan event was MS/MS in ion trap for the inclusion list of peptides representing the carotenoid proteins and internal control proteins (Thermo Application note 500; Hewel et al., 2013). A retention time (RT) window tolerance of RT±2 was used with an isolation width of one. Ions detected in the defined RT window were isolated and fragmented with a normalised collision energy of 35%. The AGC targets for Orbitrap and ion trap analysers for SIM scans were set at 100,000 and 10,000 respectively. Three biological replicates were run for all the samples.

The raw files obtained after TPM were analysed in PD 1.4 using the search and selection criteria as described under data analysis and in Kilambi *et al.* (2016). Each peptide was manually extracted from the .RAW file using Xcalibur software and evaluated for peak shape and retention time. The peptide identity was confirmed by matching its fragmentation spectrum with that of precursor fragmentation spectrum obtained from proteome profiles. Only those peaks that fulfilled these criteria were selected for measuring the area under the curve (AUC). For every targeted protein, three peptides were considered. The peptide with the best peak shape was quantified, and the other two were used for confirmation. Similarly, AUC values for internal reference peptides fulfilling the outlined criteria were noted and label free quantitation was performed for determining protein abundances.

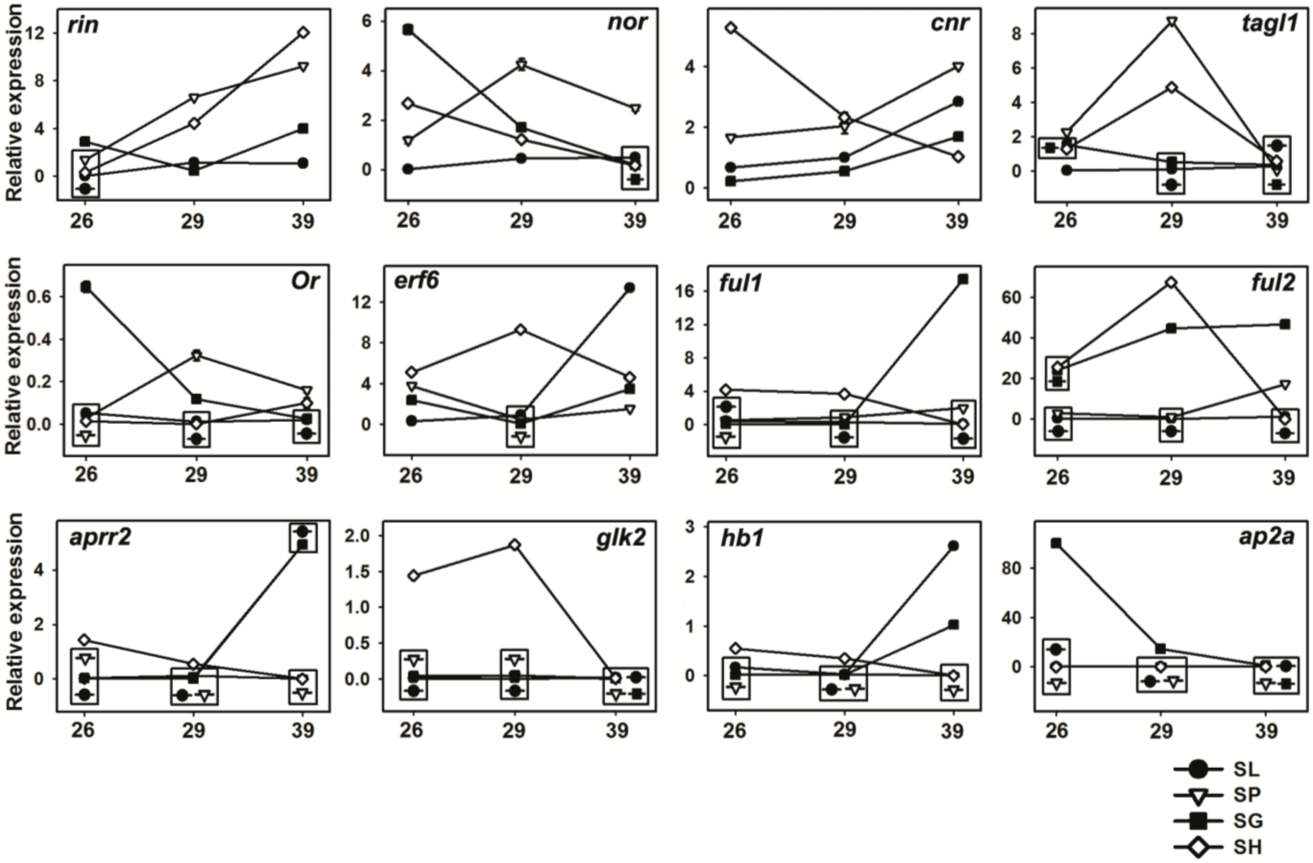

Figure S5. Relative expression levels of regulatory genes in fruits of tomato and wild species. Transcript levels were examined at 26, 29, and 39 dpa in the fruits of all genotypes. The graphs depict data obtained after normalisation with β-actin and ubiquitin (Data are means ± SE (n = 3), P ≤ 0.05 and presented in Table S7). The abbreviations are: *nor, Nonripening; rin, ripening inhibitor; cnr, Colorless nonripening; hb1, HD-Zip homeobox protein1; tagl1, tomato agamous-like 1; ap2a, apetala 2a; ful1, fruitful 1; ful2, fruitful 2; erf6, ethylene response factor 6; glk2, golden 2-like; aprr2, Arabidopsis pseudo response regulator 2-like; Or, DnaJ Cys-rich zinc finger domain-containing protein.*

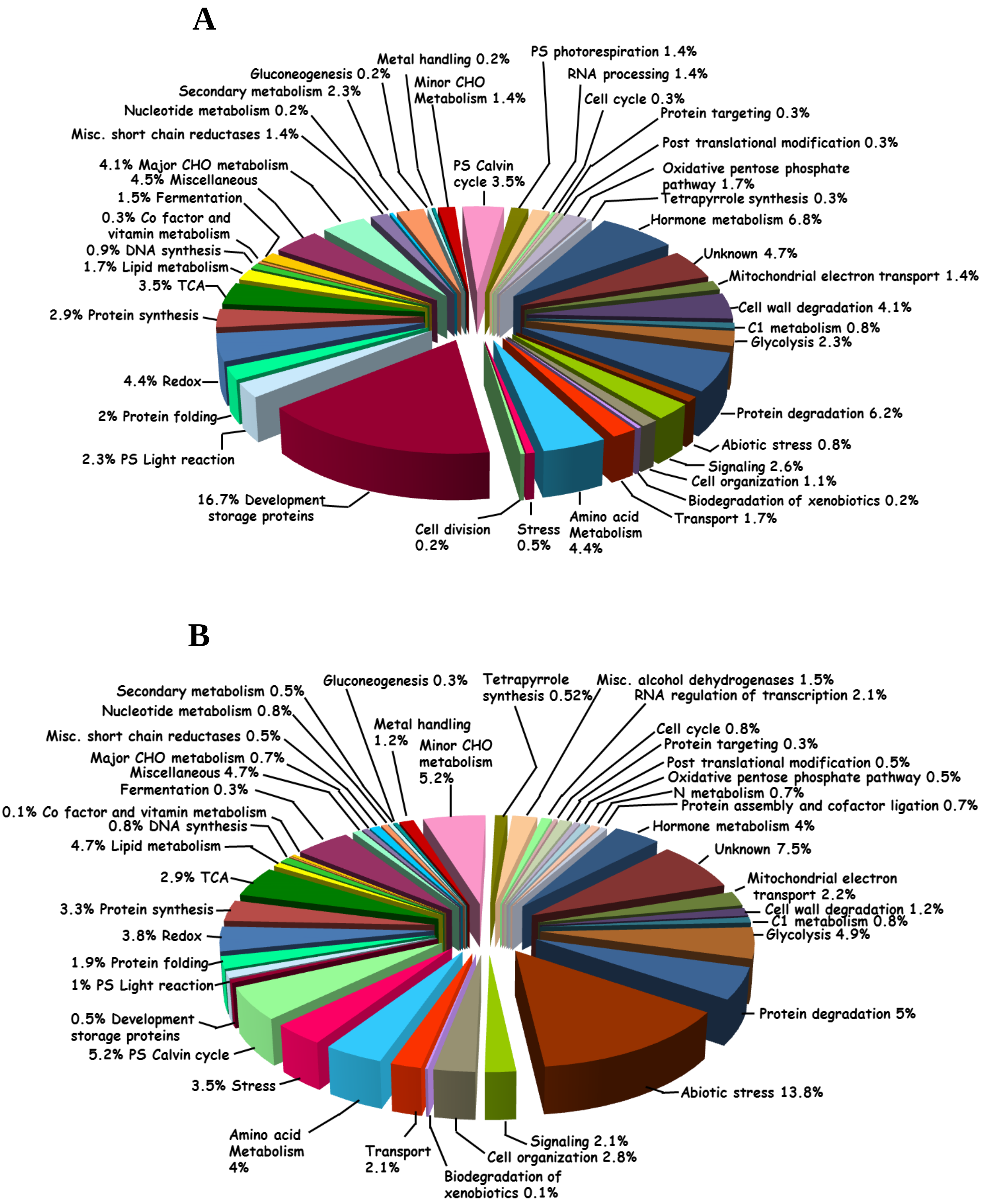
A
Metal handling 0.2%
PS photorespiration 1.4%
Gluconeogenesis 0.2%
Minor CHO Metabolism 1.4%
RNA processing 1.4%
Secondary metabolism 2.3%
Cell cycle 0.3%
Nucleotide metabolism 0.2%
Protein targeting 0.3%
Misc. short chain reductases 1.4%
Post translational modification 0.3%
4.1% Major CHO metabolism
PS Calvin cycle 3.5%
Oxidative pentose phosphate pathway 1.7%
4.5% Miscellaneous
1.5% Fermentation
Tetrapyrrole synthesis 0.3%
0.3% Co factor and vitamin metabolism
Hormone metabolism 6.8%
0.9% DNA synthesis
Unknown 4.7%
1.7% Lipid metabolism
Mitochondrial electron transport 1.4%
3.5% TCA
Cell wall degradation 4.1%
2.9% Protein synthesis
C1 metabolism 0.8%
Glycolysis 2.3%
4.4% Redox
Protein degradation 6.2%
2% Protein folding
Abiotic stress 0.8%
2.3% PS Light reaction
Signaling 2.6%
Cell organization 1.1%
Biodegradation of xenobiotics 0.2%
16.7% Development storage proteins
Transport 1.7%
Amino acid Metabolism 4.4%
Cell division 0.2%
Stress 0.5%
B
Gluconeogenesis 0.3%
Tetrapyrrole synthesis 0.52%
Misc. alcohol dehydrogenases 1.5%
Secondary metabolism 0.5%
RNA regulation of transcription 2.1%
Nucleotide metabolism 0.8%
Metal handling 1.2%
Cell cycle 0.8%
Misc. short chain reductases 0.5%
Minor CHO metabolism 5.2%
Protein targeting 0.3%
Major CHO metabolism 0.7%
Post translational modification 0.5%
Miscellaneous 4.7%
Oxidative pentose phosphate pathway 0.5%
Fermentation 0.3%
N metabolism 0.7%
Protein assembly and cofactor ligation 0.7%
0.1% Co factor and vitamin metabolism
0.8% DNA synthesis
Hormone metabolism 4%
4.7% Lipid metabolism
Unknown 7.5%
2.9% TCA
Mitochondrial electron transport 2.2%
3.3% Protein synthesis
Cell wall degradation 1.2%
C1 metabolism 0.8%
3.8% Redox
Glycolysis 4.9%
1.9% Protein folding
1% PS Light reaction
Protein degradation 5%
0.5% Development storage proteins
5.2% PS Calvin cycle
3.5% Stress
Abiotic stress 13.8%
Amino acid Metabolism 4%
Signaling 2.1%
Cell organization 2.8%
Transport 2.1%
Biodegradation of xenobiotics 0.1%

Figure S6

C

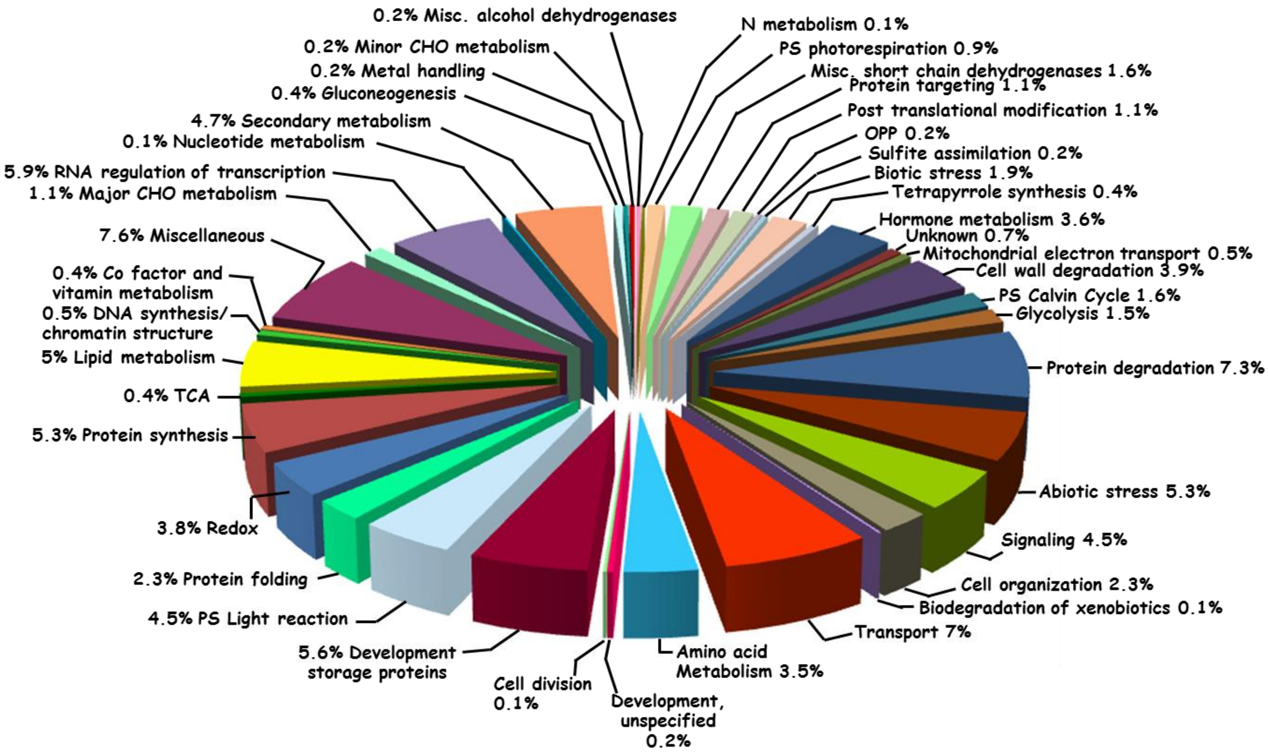
0.2% Misc. alcohol dehydrogenases
0.2% Minor CHO metabolism
0.2% Metal handling
0.4% Gluconeogenesis
4.7% Secondary metabolism
0.1% Nucleotide metabolism
5.9% RNA regulation of transcription
1.1% Major CHO metabolism
7.6% Miscellaneous
0.4% Co factor and vitamin metabolism
0.5% DNA synthesis/ chromatin structure
5% Lipid metabolism
0.4% TCA
5.3% Protein synthesis
3.8% Redox
2.3% Protein folding
4.5% PS Light reaction
5.6% Development storage proteins
Cell division 0.1%
Development, unspecified 0.2%
Amino acid Metabolism 3.5%
Transport 7%
Biodegradation of xenobiotics 0.1%
Cell organization 2.3%
Signaling 4.5%
Abiotic stress 5.3%
Protein degradation 7.3%
Glycolysis 1.5%
PS Calvin Cycle 1.6%
Cell wall degradation 3.9%
Mitochondrial electron transport 0.5%
Unknown 0.7%
Hormone metabolism 3.6%
Tetrapyrrole synthesis 0.4%
Biotic stress 1.9%
Sulfite assimilation 0.2%
OPP 0.2%
Post translational modification 1.1%
Protein targeting 1.1%
Misc. short chain dehydrogenases 1.6%
PS photorespiration 0.9%
N metabolism 0.1%

D

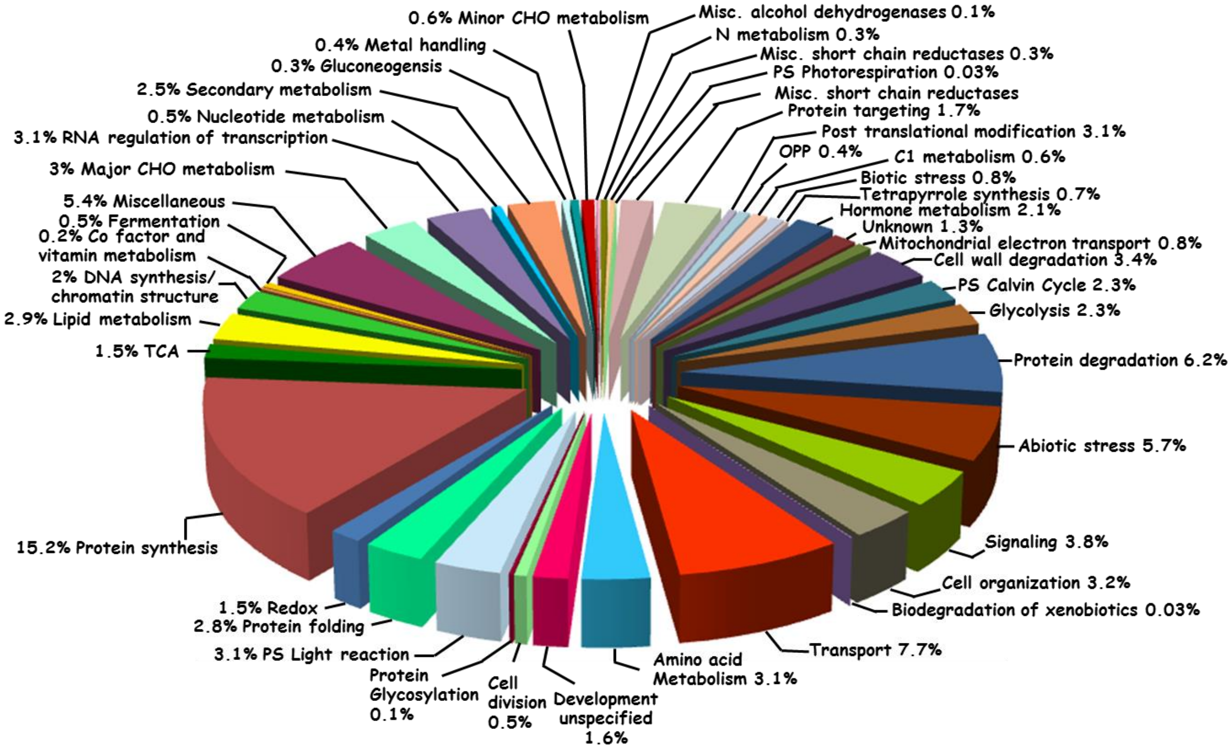
0.6% Minor CHO metabolism
0.4% Metal handling
0.3% Gluconeogensis
2.5% Secondary metabolism
0.5% Nucleotide metabolism
3.1% RNA regulation of transcription
3% Major CHO metabolism
5.4% Miscellaneous
0.5% Fermentation
0.2% Co factor and vitamin metabolism
2% DNA synthesis/ chromatin structure
2.9% Lipid metabolism
1.5% TCA
15.2% Protein synthesis
1.5% Redox
2.8% Protein folding
3.1% PS Light reaction
Protein Glycosylation 0.1%
Cell division 0.5%
Development unspecified 1.6%
Amino acid Metabolism 3.1%
Transport 7.7%
Biodegradation of xenobiotics 0.03%
Cell organization 3.2%
Signaling 3.8%
Abiotic stress 5.7%
Protein degradation 6.2%
Glycolysis 2.3%
PS Calvin Cycle 2.3%
Cell wall degradation 3.4%
Mitochondrial electron transport 0.8%
Unknown 1.3%
Hormone metabolism 2.1%
Tetrapyrrole synthesis 0.7%
Biotic stress 0.8%
C1 metabolism 0.6%
OPP 0.4%
Post translational modification 3.1%
Protein targeting 1.7%
Misc. short chain reductases
PS Photorespiration 0.03%
Misc. short chain reductases 0.3%
N metabolism 0.3%
Misc. alcohol dehydrogenases 0.1%

Figure S6

E

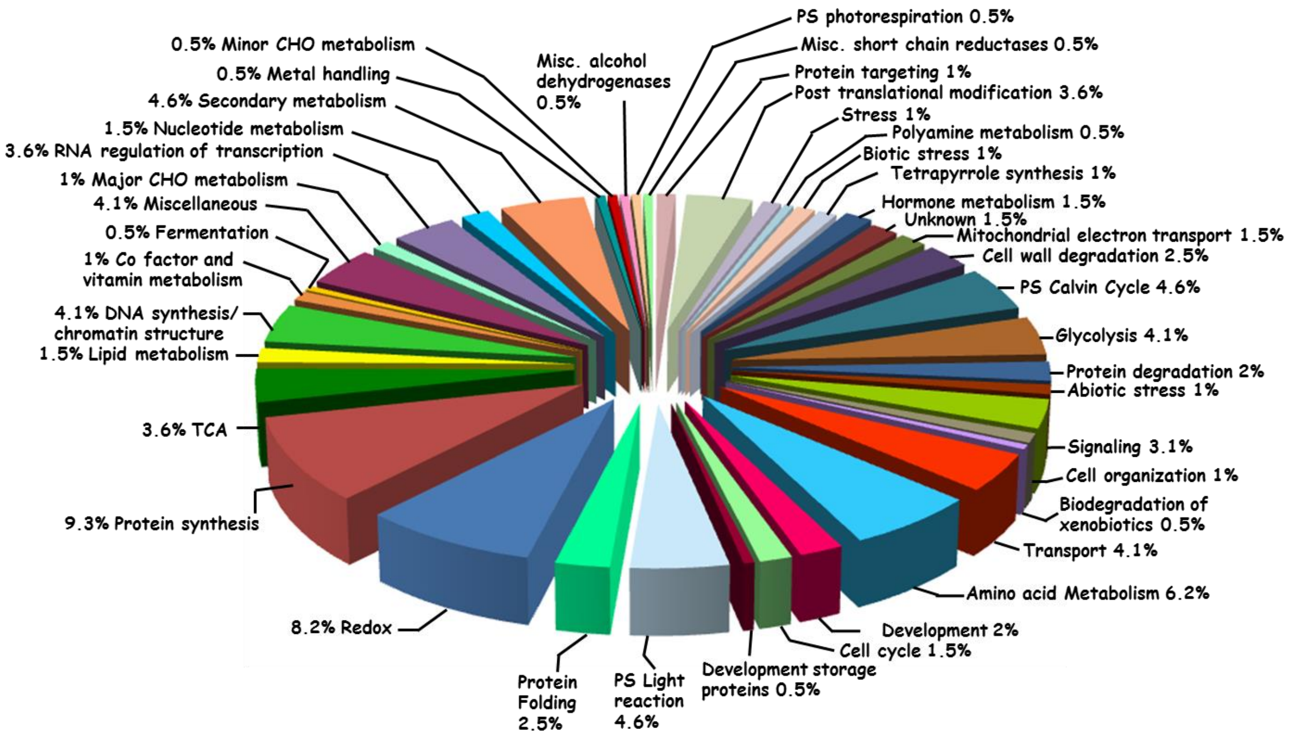
PS photorespiration 0.5%
Misc. short chain reductases 0.5%
Protein targeting 1%
Post translational modification 3.6%
Stress 1%
Polyamine metabolism 0.5%
Biotic stress 1%
Tetrapyrrole synthesis 1%
Hormone metabolism 1.5%
Unknown 1.5%
Mitochondrial electron transport 1.5%
Cell wall degradation 2.5%
PS Calvin Cycle 4.6%
Glycolysis 4.1%
Protein degradation 2%
Abiotic stress 1%
Signaling 3.1%
Cell organization 1%
Biodegradation of xenobiotics 0.5%
Transport 4.1%
Amino acid Metabolism 6.2%
Development 2%
Cell cycle 1.5%
Development storage proteins 0.5%
PS Light reaction 4.6%
Protein Folding 2.5%
8.2% Redox
9.3% Protein synthesis
3.6% TCA
1.5% Lipid metabolism
4.1% DNA synthesis/ chromatin structure
1% Co factor and vitamin metabolism
0.5% Fermentation
4.1% Miscellaneous
1% Major CHO metabolism
3.6% RNA regulation of transcription
1.5% Nucleotide metabolism
4.6% Secondary metabolism
0.5% Metal handling
0.5% Minor CHO metabolism
Misc. alcohol dehydrogenases 0.5%

F

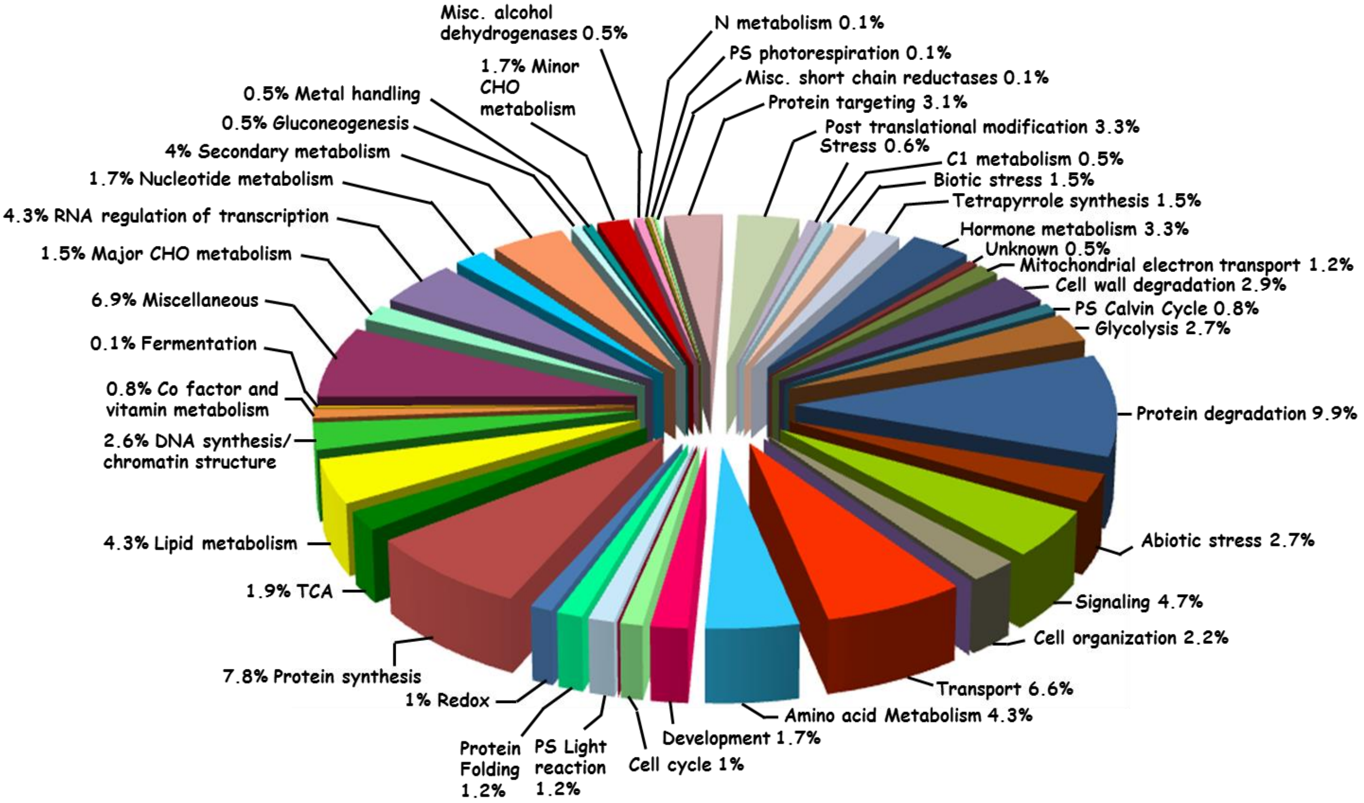
Misc. alcohol dehydrogenases 0.5%
N metabolism 0.1%
PS photorespiration 0.1%
Misc. short chain reductases 0.1%
Protein targeting 3.1%
Post translational modification 3.3%
Stress 0.6%
C1 metabolism 0.5%
Biotic stress 1.5%
Tetrapyrrole synthesis 1.5%
Hormone metabolism 3.3%
Unknown 0.5%
Mitochondrial electron transport 1.2%
Cell wall degradation 2.9%
PS Calvin Cycle 0.8%
Glycolysis 2.7%
Protein degradation 9.9%
Abiotic stress 2.7%
Signaling 4.7%
Cell organization 2.2%
Transport 6.6%
Amino acid Metabolism 4.3%
Development 1.7%
Cell cycle 1%
PS Light reaction 1.2%
Protein Folding 1.2%
1% Redox
7.8% Protein synthesis
1.9% TCA
4.3% Lipid metabolism
2.6% DNA synthesis/ chromatin structure
0.8% Co factor and vitamin metabolism
0.1% Fermentation
6.9% Miscellaneous
1.5% Major CHO metabolism
4.3% RNA regulation of transcription
1.7% Nucleotide metabolism
4% Secondary metabolism
0.5% Gluconeogenesis
0.5% Metal handling
1.7% Minor CHO metabolism

Figure S6

Figure S6. Functional classification of differentially expressed proteins in the fruits of wild species. The differentially regulated proteins in the fruits of wild species compared to SL were functionally classified using MapMan. These proteins are also listed in Table S4. **A,** SP-SL-upregulated proteins; **B,** SP-SL-downregulated proteins; **C,** SG-SL-upregulated proteins; **D,** SG-SL-downregulated proteins; E**,** SH-SL-upregulated proteins; **F,** SH-SL-downregulated proteins.

A

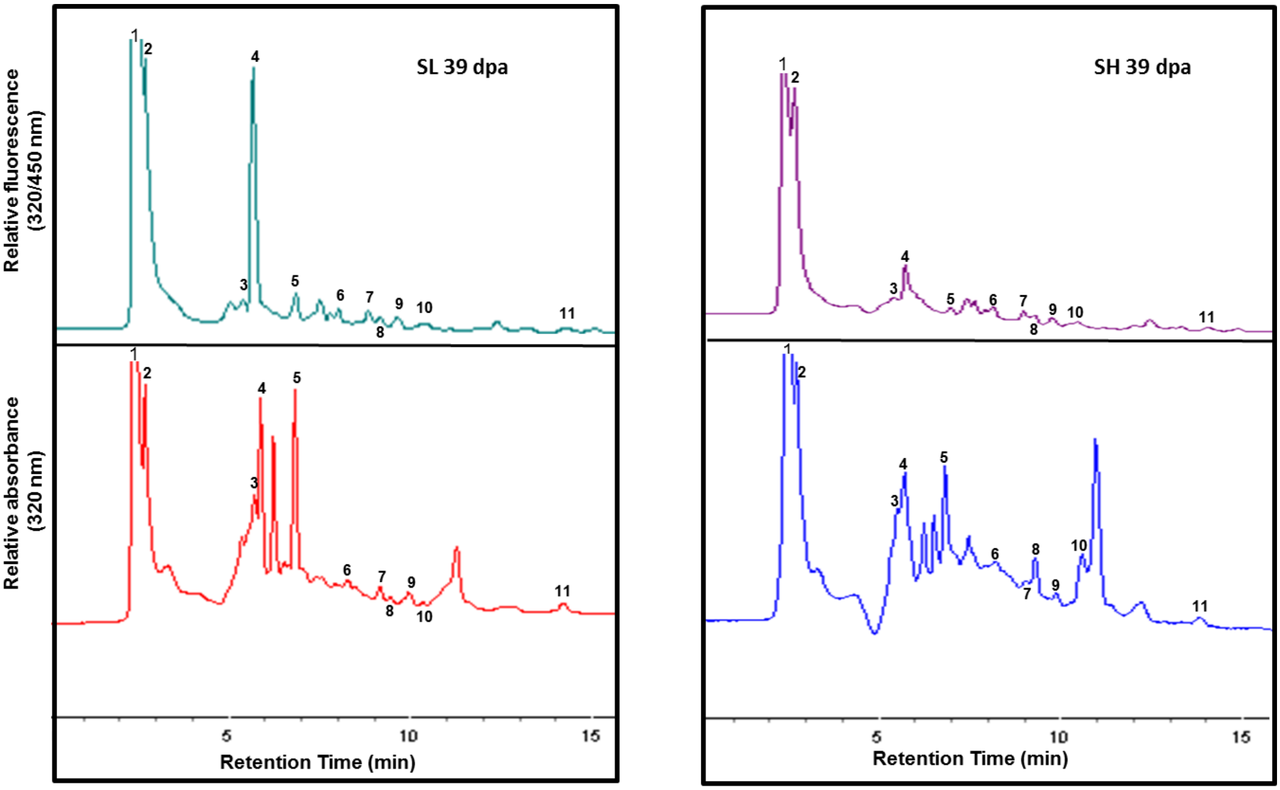

SL 39 dpa
SH 39 dpa
Relative fluorescence (320/450 nm)
Relative absorbance (320 nm)
Retention Time (min)
1
2
3
4
5
6
7
8
9
10
11
5
10
15

Figure S7.

B

Peak No.	Mean Peak area in SL± S.E. (Fluorescence at 320/450 nm)	Mean Peak area in SH± S.E. (Fluorescence at 320/450 nm)	Ratio (SL/SH)	p value	Mean Peak area in SL		Mean Peak area in SH	
					$A_{320\ nm}$	$A_{360\ nm}$	$A_{320\ nm}$	$A_{360\ nm}$
1	8667988.67± 2167647	1299059.66± 187294	6.67	p<0.027	371195.67± 32514	24000± 22345	148478.27± 13005	96700
2	4527947.67± 105534	2069653.33± 373448	2.19	p<0.03	163954.67± 21774	51200± 3456	117110.48± 15553	40000
3	506793.33± 84606	230826.66± 10629	2.20	p<0.03	89920± 1483	123500± 43542	32114.29± 530	45600
4	2271667.67± 257265	461659.33± 47956	4.92	p<0.002	83113.33± 1288	NA	15983.33± 248	NA
5	429221.67± 38624	76461± 2549	5.61	p<0.0008	153863± 19106	NA	28493.15± 3538	NA
6	305893± 25179	85974± 4899	3.56	p<0.001	28661.67± 726	NA	8685.35± 220	NA
7	384425.33± 25827	100407.33± 2418	3.83	p<0.0003	23607± 1097	NA	6053± 281	NA
8	226343± 18026	81262.33± 7793	2.79	p<0.001	15786± 1527	NA	6071.54± 587	NA
9	191336 ± 10048	55701.66± 2362	3.44	p<0.0001	25416.33± 1742	NA	7060± 484	NA
10	191292.66 ± 14444	44768.33± 741	4.28	p<0.0005	9642.33± 321	NA	2351.79± 78	NA
11	106210± 14444	23595± 1161	4.50	p<0.00004	16487.33± 2031	NA	4021.30± 496	NA

Figure S7. A, HPLC traces showing the absorbance and fluorescence spectra and **B,** peak areas of primary fluorescence catabolites (pFCCs) obtained after PAO/RCCR assay in 39 dpa fruit extracts of SL and SH. Data are means ± SE (n = 3), $P \leq 0.05$.

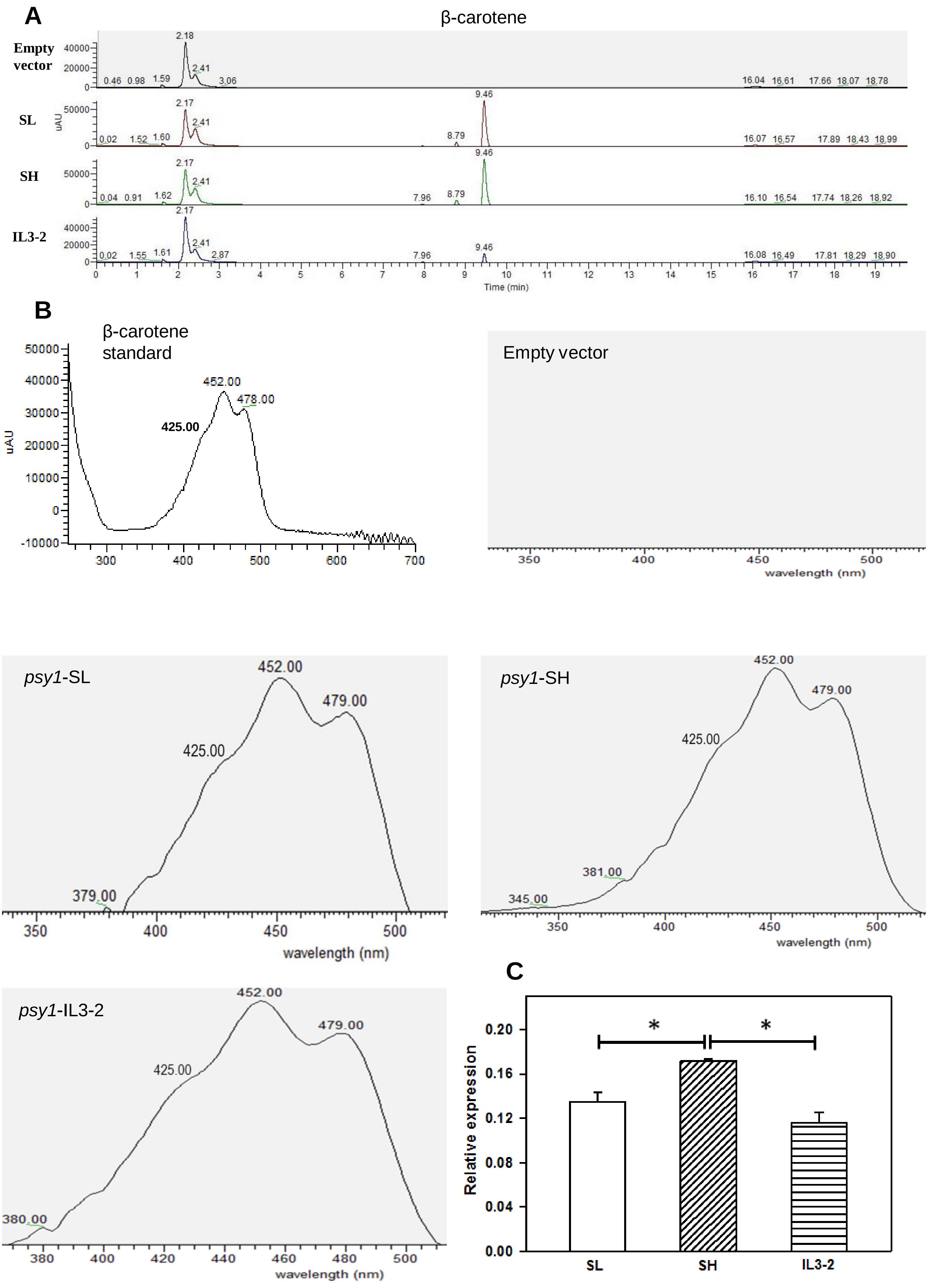

A
β-carotene
Empty vector
SL
SH
IL3-2
uAU
Time (min)
B
β-carotene standard
Empty vector
psy1-SL
psy1-SH
psy1-IL3-2
wavelength (nm)
C
Relative expression
SL
SH
IL3-2

Figure S8

D

SNPs in *psy1* (Solyc03g031860.2.1)					
Solanum lycopersicum		***Solanum habrochaites***		***Solanum pennellii***	
**A.A change**	**SIFT score**	**A.A change**	**SIFT score**	**A.A change**	**SIFT score**
		Q95Q		G71G	
		R158R		R158R	
		P167P		P167P	
		N189N		V319V	
		T246T		V345V	
		L249L		A408V	0.274
		A307A			
		Y363Y			
		A408V	0.274		

E

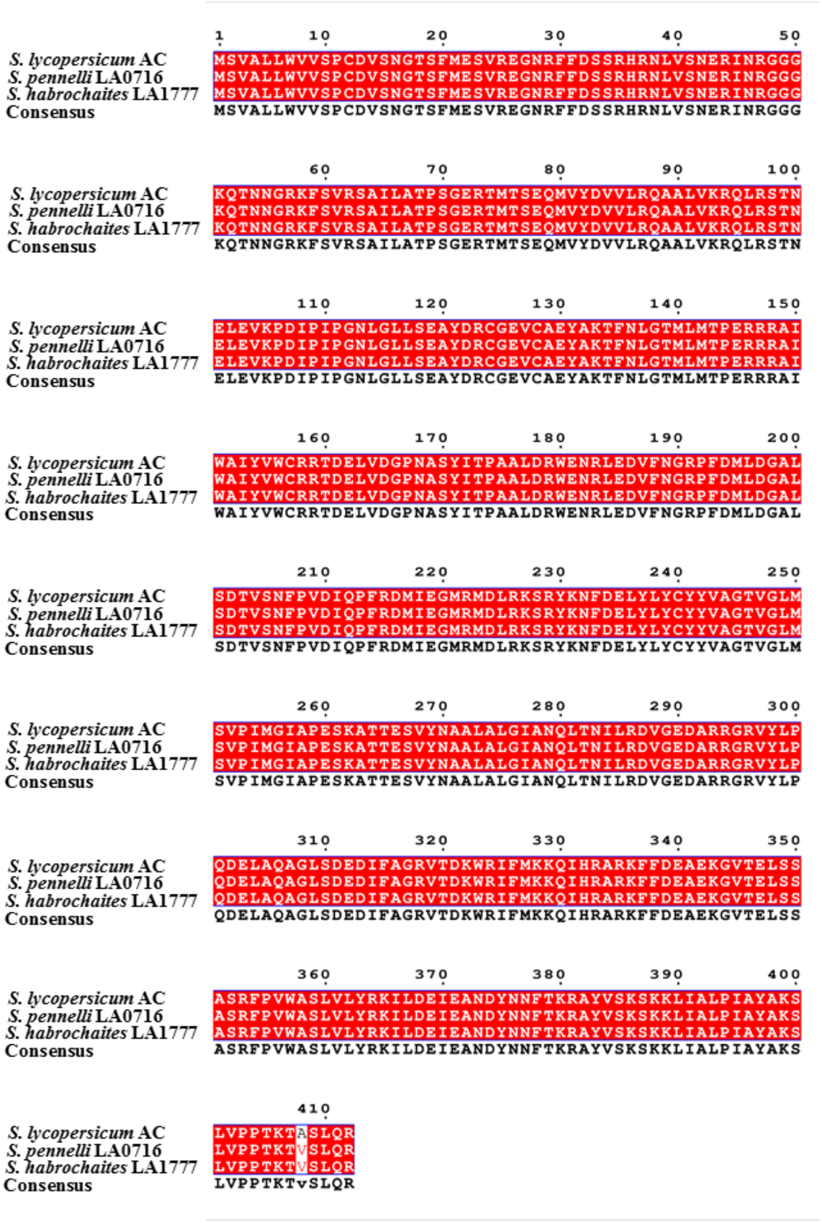

Figure S8

F

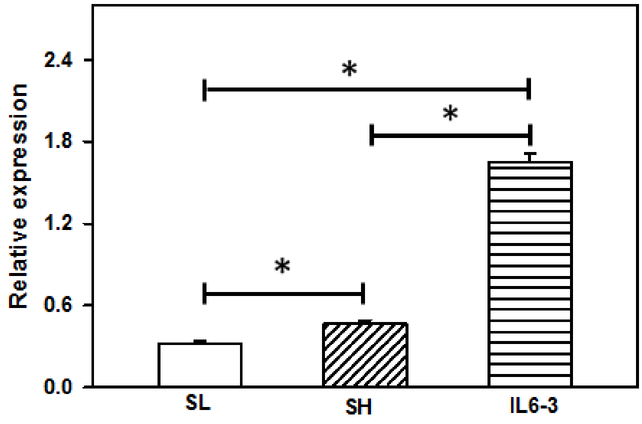

G

SNPs in *cycb* (Solyc06g074240.1.1)					
Solanum lycopersicum		***Solanum habrochaites***		***Solanum pennellii***	
**A.A change**	**SIFT score**	**A.A change**	**SIFT score**	**A.A change**	**SIFT score**
		V23F	0.734	T3A	0.018
		P27P		Y19H	0.546
		L115L		V23F	0.734
		K153K		P32P	
		N175N		K35Q	0.429
		R229K	1	S77S	
		E244D	0.012	N78G	0.63
		L265L		L95L	
		V304V		K153K	
		V335L	1	N175N	
		L484V	1	R229K	1
				L265L	
				D290N	1
				V304V	
				K326R	0.36
				T363T	
				N472N	
				M473L	0.38
				L484V	1
				I495V	1

Figure S8

H

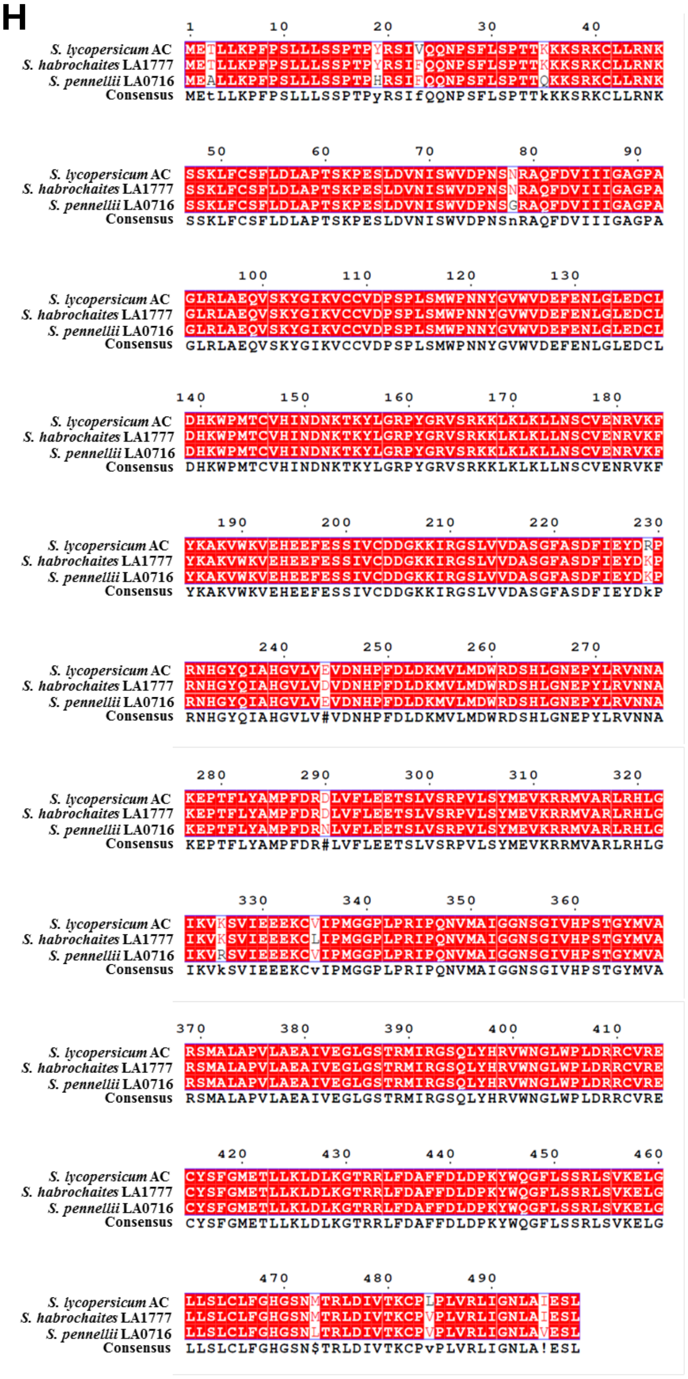

S. lycopersicum AC METLLKPFPSLLLSSPTPYRSIVQQNPSFLSPTTKKKSRKCLLRNK
S. habrochaites LA1777 METLLKPFPSLLLSSPTPYRSIFQQNPSFLSPTTKKKSRKCLLRNK
S. pennellii LA0716 MEALLKPFPSLLLSSPTPHRSIFQQNPSFLSPTTQKKSRKCLLRNK
Consensus MEtLLKPFPSLLLSSPTPyRSIfQQNPSFLSPTTkKKSRKCLLRNK
S. lycopersicum AC SSKLFCSFLDLAPTSKPESLDVNISWVDPNSNRAQFDVIIIGAGPA
S. habrochaites LA1777 SSKLFCSFLDLAPTSKPESLDVNISWVDPNSNRAQFDVIIIGAGPA
S. pennellii LA0716 SSKLFCSFLDLAPTSKPESLDVNISWVDPNSGRAQFDVIIIGAGPA
Consensus SSKLFCSFLDLAPTSKPESLDVNISWVDPNSnRAQFDVIIIGAGPA
S. lycopersicum AC GLRLAEQVSKYGIKVCCVDPSPLSMWPNNYGVWVDEFENLGLEDCL
S. habrochaites LA1777 GLRLAEQVSKYGIKVCCVDPSPLSMWPNNYGVWVDEFENLGLEDCL
S. pennellii LA0716 GLRLAEQVSKYGIKVCCVDPSPLSMWPNNYGVWVDEFENLGLEDCL
Consensus GLRLAEQVSKYGIKVCCVDPSPLSMWPNNYGVWVDEFENLGLEDCL
S. lycopersicum AC DHKWPMTCVHINDNKTKYLGRPYGRVSRKKLKLKLLNSCVENRVKF
S. habrochaites LA1777 DHKWPMTCVHINDNKTKYLGRPYGRVSRKKLKLKLLNSCVENRVKF
S. pennellii LA0716 DHKWPMTCVHINDNKTKYLGRPYGRVSRKKLKLKLLNSCVENRVKF
Consensus DHKWPMTCVHINDNKTKYLGRPYGRVSRKKLKLKLLNSCVENRVKF
S. lycopersicum AC YKAKVWKVEHEEFESSIVCDDGKKIRGSLVVDASGFASDFIEYDRP
S. habrochaites LA1777 YKAKVWKVEHEEFESSIVCDDGKKIRGSLVVDASGFASDFIEYDKP
S. pennellii LA0716 YKAKVWKVEHEEFESSIVCDDGKKIRGSLVVDASGFASDFIEYDKP
Consensus YKAKVWKVEHEEFESSIVCDDGKKIRGSLVVDASGFASDFIEYDkP
S. lycopersicum AC RNHGYQIAHGVLVEVDNHPFDLDKMVLMDWRDSHLGNEPYLRVNNA
S. habrochaites LA1777 RNHGYQIAHGVLVDVDNHPFDLDKMVLMDWRDSHLGNEPYLRVNNA
S. pennellii LA0716 RNHGYQIAHGVLVEVDNHPFDLDKMVLMDWRDSHLGNEPYLRVNNA
Consensus RNHGYQIAHGVLV#VDNHPFDLDKMVLMDWRDSHLGNEPYLRVNNA
S. lycopersicum AC KEPTFLYAMPFDRDLVFLEETSLVSRPVLSYMEVKRRMVARLRHLG
S. habrochaites LA1777 KEPTFLYAMPFDRDLVFLEETSLVSRPVLSYMEVKRRMVARLRHLG
S. pennellii LA0716 KEPTFLYAMPFDRNLVFLEETSLVSRPVLSYMEVKRRMVARLRHLG
Consensus KEPTFLYAMPFDR#LVFLEETSLVSRPVLSYMEVKRRMVARLRHLG
S. lycopersicum AC IKVKSVIEEEKCVIPMGGPLPRIPQNVMAIGGNSGIVHPSTGYMVA
S. habrochaites LA1777 IKVKSVIEEEKCLIPMGGPLPRIPQNVMAIGGNSGIVHPSTGYMVA
S. pennellii LA0716 IKVRSVIEEEKCVIPMGGPLPRIPQNVMAIGGNSGIVHPSTGYMVA
Consensus IKVkSVIEEEKCvIPMGGPLPRIPQNVMAIGGNSGIVHPSTGYMVA
S. lycopersicum AC RSMALAPVLAEAIVEGLGSTRMIRGSQLYHRVWNGLWPLDRRCVRE
S. habrochaites LA1777 RSMALAPVLAEAIVEGLGSTRMIRGSQLYHRVWNGLWPLDRRCVRE
S. pennellii LA0716 RSMALAPVLAEAIVEGLGSTRMIRGSQLYHRVWNGLWPLDRRCVRE
Consensus RSMALAPVLAEAIVEGLGSTRMIRGSQLYHRVWNGLWPLDRRCVRE
S. lycopersicum AC CYSFGMETLLKLDLKGTRRLFDAFFDLDPKYWQGFLSSRLSVKELG
S. habrochaites LA1777 CYSFGMETLLKLDLKGTRRLFDAFFDLDPKYWQGFLSSRLSVKELG
S. pennellii LA0716 CYSFGMETLLKLDLKGTRRLFDAFFDLDPKYWQGFLSSRLSVKELG
Consensus CYSFGMETLLKLDLKGTRRLFDAFFDLDPKYWQGFLSSRLSVKELG
S. lycopersicum AC LLSLCLFGHGSNMTRLDIVTKCPLPLVRLIGNLAIESL
S. habrochaites LA1777 LLSLCLFGHGSNMTRLDIVTKCPVPLVRLIGNLAIESL
S. pennellii LA0716 LLSLCLFGHGSNLTRLDIVTKCPVPLVRLIGNLAVESL
Consensus LLSLCLFGHGSN$TRLDIVTKCPvPLVRLIGNLA!ESL

Figure S8

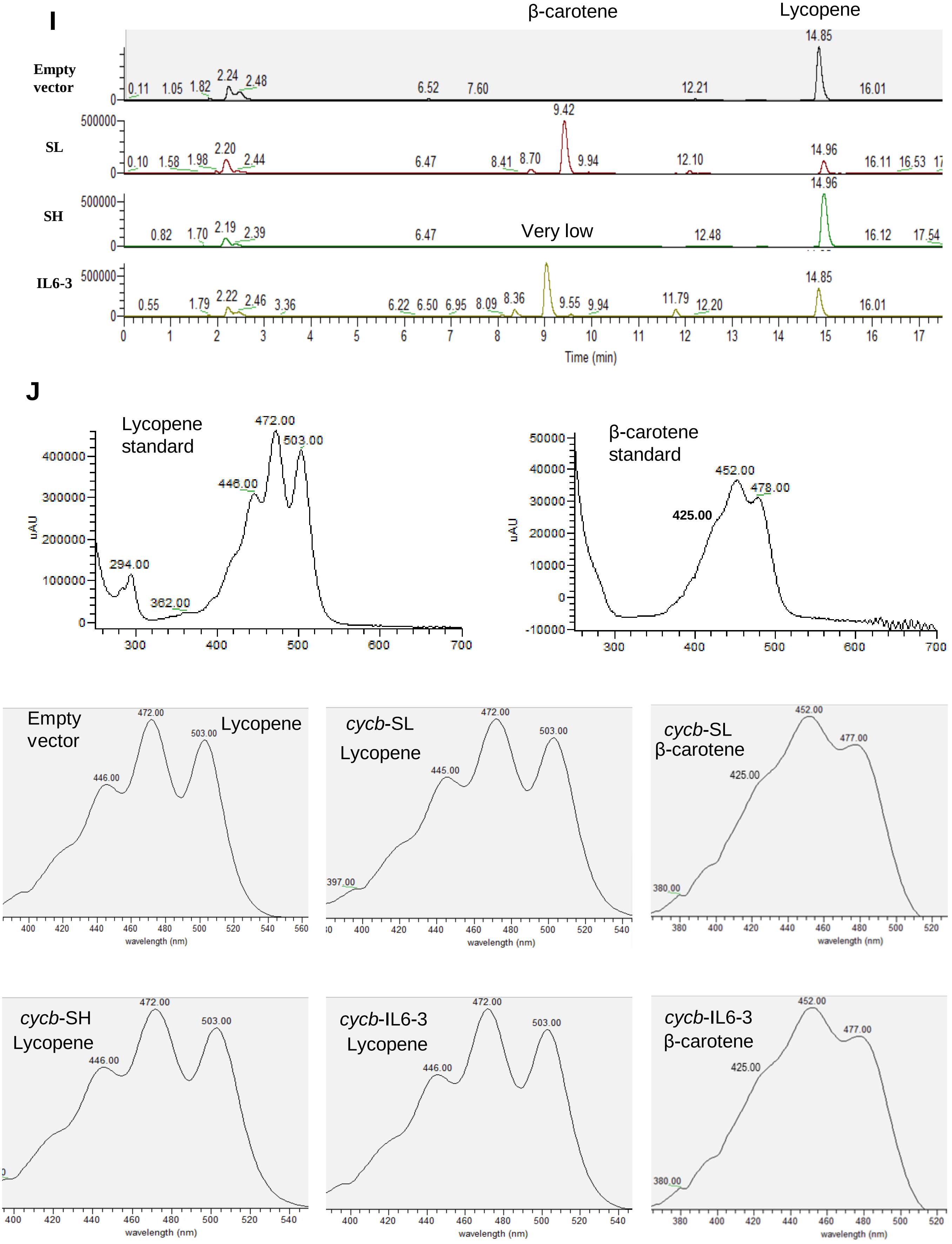
I
β-carotene
Lycopene
Empty vector
SL
SH
IL6-3
Very low
Time (min)
J
Lycopene standard
β-carotene standard
Empty vector Lycopene
cycb-SL Lycopene
cycb-SL β-carotene
cycb-SH Lycopene
cycb-IL6-3 Lycopene
cycb-IL6-3 β-carotene
wavelength (nm)

Figure S8

Figure S8. Functional complementation of *psy1* and *cycb* in *E. coli*. *E. coli* having pAC-85b plasmid was transfected with pET32a constructs with *psy1* from SL, SH and IL3-2. Similarly, *E. coli* with pAC-LYC plasmid was transfected with pET32a constructs with *cycb* from SL, SH and IL6-3. Carotenoids were extracted and analysed on UHPLC as described in Gupta *et al.* 2015. (Data are means ± SE (*n* = 3), P ≤ 0.05). **A**, Elution profiles of cell extracts transformed with empty vector, vector with *psy1*-SL, *psy1*-SH and *psy1*-IL3-2 constructs. **B**, Extracted PDA spectra for the β-carotene peak. **C**, Relative expression of *psy1* gene after normalization with *actin* and *ubiquitin* (Data are means ± SE (n = 3), * = P ≤ 0.05). **D**, List of SNPs (retrieved from JBROWSE (http://www.tomatogenome.net/VariantBrowser, Aflitos *et al.* 2014 and SGN (http://solgenomics.net/) in *psy1* from SL, SH and *S. pennellii* and SIFT scores (Vaser *et al.* 2016). **E**, Amino acid sequence alignment of PSY1 from SL, SH and *S. pennellii*, another green-fruited wild species. Note the presence of single amino acid substitution, A408V in both SH and *S. pennellii*. **F**, Relative expression of *cycb* gene after normalization with *actin* and *ubiquitin* (Data are means ± SE (n = 3), * = P ≤ 0.05). **G**, List of SNPs in *cycb* from SL, SH and *S. pennellii* and SIFT scores. **H**, Amino acid sequence alignment of CYCB from SL, SH and *S. pennellii*. Note the presence of multiple amino acid substitutions in both SH and *S. pennellii*. **I**, Elution profiles of cell extracts transformed with empty vector, vector with *cycb*-SL, *cycb*-SH and *cycb*-IL6-3 constructs. **J**, Extracted PDA spectra for the lycopene and β-carotene peaks.
